# Exploring the Relationship between Dietary Intake and Clinical Outcomes in Peritoneal Dialysis Patients Stratified by Serum Albumin Levels: A 12-Year Follow-Up Using Fine-Grained Electronic Medical Records Data

**DOI:** 10.34133/hds.0280

**Published:** 2025-07-02

**Authors:** Yueying Wu, Junyi Gao, Wen Tang, Chunyan Su, Yinghao Zhu, Tianlong Wang, Ling Wang, Weibin Liao, Xu Chu, Yasha Wang, Xinju Zhao, Tao Wang, Ewen M. Harrison, Liantao Ma

**Affiliations:** ^1^ Peking University, Beijing, China.; ^2^ Health Data Research and University of Edinburgh, Edinburgh, UK.; ^3^ Department of Nephrology, Peking University Third Hospital, Beijing, China.; ^4^ Department of Nephrology, the Affiliated Xuzhou Municipal Hospital of Xuzhou Medical University, Xuzhou, China.; ^5^ Tsinghua University, Beijing, China.; ^6^ Peking University People’s Hospital, Beijing, China.; ^7^ University of Edinburgh, Edinburgh, UK.

## Abstract

End-stage renal disease (ESRD) significantly impacts patients’ quality of life and poses substantial socioeconomic burdens. Dietary interventions are crucial for managing ESRD, yet high-quality evidence and analysis specifically linking diet to mortality outcomes is scarce. **Methods:** We conducted a comprehensive study involving 656 peritoneal dialysis (PD) patients over 12 years, with an average follow-up every 3 months. Dietary intake was meticulously recorded using a 3-day dietary record method, integrated with detailed health records and outcomes. We employed a 2-stage model to evaluate nonlinear relationships between dietary nutrients and mortality risk, accounting for various confounding factors. **Findings:** Our analysis revealed that 14 out of 26 nutritional elements lack guidelines for ESRD and PD patients, with 13 showing significant associations with mortality. For example, while guidelines suggest a dietary protein intake of 1.0 to 1.2 g/kg/d, our findings indicate an optimal range of 0.88 to 1.13 g/kg/d. Similarly, the recommended dietary energy intake of 25 to 35 kcal/kg/d was refined to 26 to 42 kcal/kg/d. We identified that 69% of dietary intake–outcome relationships are nonlinear, especially in patients with poor health status. **Interpretations:** Our study provides detailed dietary intake thresholds that correlate with improved prognosis in ESRD patients, enhancing current guidelines. The findings highlight the importance of personalized nutritional management and underscore the nonlinear nature of nutrient–disease relationships, particularly in severely ill patients. This approach can refine dietary recommendations and improve patient care in ESRD.

## Introduction

End-stage renal disease (ESRD) represents a significant global health challenge, profoundly impacting patients’ lives through both the disease’s direct effects and the substantial socioeconomic costs associated with management and treatment [1]. Patients with ESRD experience a diminished quality of life, grappling with the burdens of chronic illness and the rigors of treatment regimens, including peritoneal dialysis (PD) [2]. Given the chronic nature of ESRD and the reliance on dialysis, dietary interventions emerge as crucial for slowing disease progression and preventing adverse outcomes, underscoring the importance of tailored nutritional guidance in this context [3,4]. Studies have confirmed that dietary interventions through digital health approaches can effectively manage blood glucose levels in diabetic patients [5], a significant portion of whom have ESRD. This demonstrates the effectiveness of incorporating these dietary interventions into patient treatment plans.

The current landscape of research concerning dietary and nutritional management in ESRD often features evidence of a lower grade, typically categorized as level C, which is typically based on expert opinion, case reports, or descriptive studies, rather than randomized controlled trials or large-scale observational studies, indicating a lower quality of evidence, or based on expert opinion [6]. Most studies tend to focus on individuals with chronic kidney disease (CKD) and aim to track the progression to ESRD [7–11] and associated comorbidities [12]. However, there is a noticeable scarcity of studies specifically examining the impact of diet on mortality rates among ESRD patients. While existing studies have provided valuable information using dietary data collected from single follow-ups or patient questionnaires to track broad dietary components such as protein and energy intake [7,8,12], the detail and frequency of data collection could be enhanced to enrich the evidence base. Besides, current findings tend to present linear outcomes and might benefit from a more detailed exploration of dietary intake ranges tailored to patient needs [7]. Addressing these opportunities for refinement could advance our understanding of the relationship between diet and prognosis in ESRD, ultimately leading to more robust and clinically applicable dietary guidelines that improve patient care.

However, studying the relationship between dietary intake and prognosis indeed presents significant challenges:

• **The difficulty of collecting long-term, high-quality, and linked data.** During the prolonged progression of ESRD, both patients’ condition and dietary habits are likely to change, necessitating continuous, detailed monitoring of both disease progression and dietary intake. The dietary record should also be as granular as possible to achieve accurate and comprehensive diet planning. Besides, it is essential to integrate these dietary records with comprehensive health records and outcome data to facilitate a more accurate understanding of how diet influences ESRD progression and patient outcomes. However, accurate data collection process requires expert supervision, adherence to guidelines, and conducting comprehensive face-to-face interviews, which increases the complexity of maintaining precise records [13].
• **The complexity of the relationship between dietary intakes and outcomes.** Defining a “good diet” is challenging as the progression of ESRD is inevitable, thus obscuring the clear impact of dietary habits. Numerous confounders, such as a patient’s overall health status, can influence both their dietary choices and prognosis. These factors are typically indicated by various clinical metrics. Moreover, the relationship between dietary nutrients and health outcomes may be nonlinear. Evidence indicates that both excessive and insufficient protein intake can negatively affect disease progression. Our experiments have also shown that some nonlinear methods are inadequate in capturing these complex relationships (see the “How does dietary nutrition affect the mortality rate in patients?” section). The lack of standardized approaches to identify such nonlinearity in existing studies further complicates the identification of protective dietary nutrient ranges.

To address the challenges, we have implemented robust data collection strategies and developed a 2-stage method to analyze nonlinear relationships. Our approach has successfully identified precise ranges of dietary nutrient intake that serve as protective factors in prognosis. Our contributions are as follows:

• **Data collection:** In our clinical practice, we have amassed a comprehensive dataset from over 13,000 clinical visits, involving 656 PD patients across a span of more than 12 years, with an average follow-up frequency of every 3 months. This dataset encompasses detailed real-world dietary nutrients, follow-up data, and outcomes, providing a robust foundation for our analysis. Dietary details are recorded with high precision using the guideline-recommended, expert-supervised 3-day dietary record method [14,15].
• **Method design:** We have devised a method to manage the influence of different confounding variables. By employing clinical indicators to detect patients’ health status, we group patients with similar conditions. This stratification aims to minimize the impact of confounding variables as much as possible. Additionally, to address the intricate relationship between diet and outcomes, we have developed a 2-stage model. This model evaluates mortality risk and models nonlinear nutrient-risk correlations effectively, even in complex scenarios. Table S1 outlines the advantages of our approach compared to existing methodologies.

The aim of this study is to provide dietary nutritional intake thresholds associated with a positive prognosis by exploring the relationship between dietary intake and clinical outcomes in PD patients.

## Materials and Methods

**Fig. 1.**
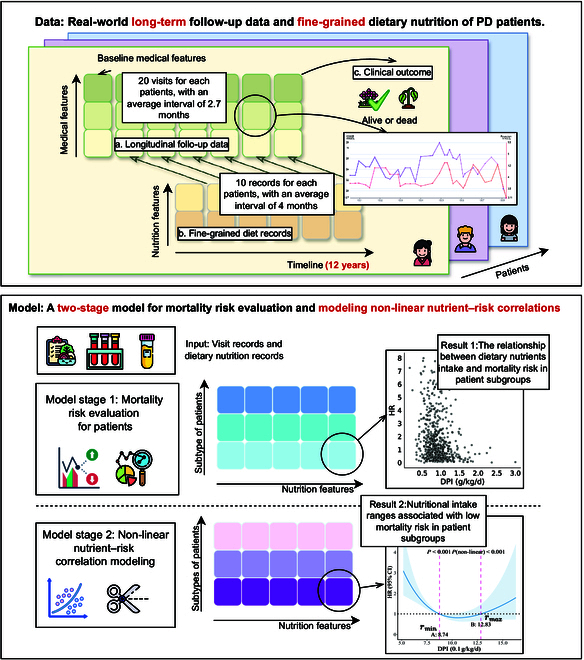
The pipeline of our method. We collected a real-world clinical EHR dataset from PD patients over 12 years, consisting of static baseline information, longitudinal multivariable records and clinical outcomes. We developed a 2-stage model to evaluate the nonlinear relationship between patients’ diet and mortality risk: (a) Prior to stage 1, we categorized patients into distinct subtypes. This stratification aids in identifying more granular patterns. Consequently, the results generated in stages 1 and 2 are grounded within specific subtypes rather than the entire patient population, allowing for more precise analysis. (b) In the first stage, we used the Cox proportional hazards model to predict patients’ mortality risk using patients’ dietary nutrition and medical examination records. The Cox model was used to assess the dietary evaluation score for each nutritional element. (c) In the second stage, we designed a nonlinear restricted cubic spline (RCS) model to process the results from the first stage. The RCS model calculates the beneficial intake range for each dietary nutrient associated with low mortality risk. The pink dashed lines in the Result 2 figures for the second stage indicate the ranges $r^{i}=\left(r_{\min}^{i},\;r_{\max}^{i}\right)$. The different shades of squares represent various patient subtypes, with each square corresponding to a specific range $r^{i}$ associated with a particular nutritional element and the corresponding patient subtype.

We designed a 2-stage model to evaluate the mortality risk for patients and to obtain precise dietary nutritional ranges associated with low mortality risk for each dietary nutrient. Figure 1 shows the pipeline of our method. In this section, we will elaborate on the problem formulation, data collection, and methodological details.

### Problem formulation

Let P denote the population of patients with PD. Our objective is to identify a daily dietary arrangement that optimizes health benefits for this group.

A beneficial dietary arrangement is characterized as one that extends the survival time T for patients with analogous baseline attributes. The survival time T is the period from the initiation of the follow-up $t_{0}$ to the end of follow-up $t_{\mathrm{end}}$, which may be due to death, loss to follow-up, or the termination of the study period.

To ensure precision and practicality in a clinical setting, the diet is quantified in terms of nutrient granularity. Let $D=\left\{d_{1},\;d_{2},\;\ldots ,\;d_{n}\right\}$ denote the collection of daily nutrient intakes for all patients, with each $d_{i}$ representing the mean dietary intake for an individual during the follow-up, inclusive of N distinct nutrients, such as water, protein, dietary fiber, copper, manganese, and so on.

The initial step involves pinpointing a dietary intake $d^{*}\in D$ that enhances the follow-up duration t to the greatest extent, denoted by the dietary evaluation score s.

The dietary benefit for a patient is assessed with the function *f*:

f:b×d×t×ω→s (1)

where

b represents the baseline characteristics of the patient, encompassing demographic markers such as gender and age, as well as clinical metrics like creatinine, albumin, blood pressure, etc.

d is the average dietary intake throughout the follow-up period.

t signifies the follow-up duration, calculated as $t=t_{\mathrm{end}}-t_{0}$.

ω denotes the outcome (e.g., alive, lost to follow-up, or deceased).

$f\left(b,\;d,\;t,\;\omega \right)$ yields the dietary evaluation score for a patient, defined as s, where s∈S.

This produces the set of diet and dietary evaluation score pairs for all patients $P=\left\{\left(d,\;s\right)|\;d\in D,\;s\in S\right\}$. By disaggregating d into its components, we obtain the collection of all patients’ records for the ith nutrient and its corresponding evaluation $P_{i}=\left\{\left(t_{i},\;s\right)\right\}$.

Proceeding to the second step, to extrapolate from individual findings to the broader population, we introduce a function g that processes the set of all patient diet–evaluation pairs and identifies the range of nutrient intakes linked to prolonged survival times:

g:t×s→r (2)

where

t is the intake of a specific dietary nutrient.

s represents the dietary evaluation score corresponding to that nutrient intake.

r denotes the intake range of the dietary nutrient associated with low mortality risk, which can extend survival times as much as possible, given by $r=\left(r_{\min},\;r_{\max}\right)$, where $r_{\min}$ denotes the minimum intake that meets the condition and $r_{\max}$ denotes the maximum one.

Hence, we determine the specific intake range for each dietary nutrient $t_{1}$ to $t_{N}$ connected with a lower mortality risk:

$$R=\left\{r^{i}=\left\{r_{\min}^{i},\;r_{\max}^{i}\right\}|\;1\leq i\leq N\right\}$$ (3)

### Data source and collection

#### Data source

**Fig. 2.**
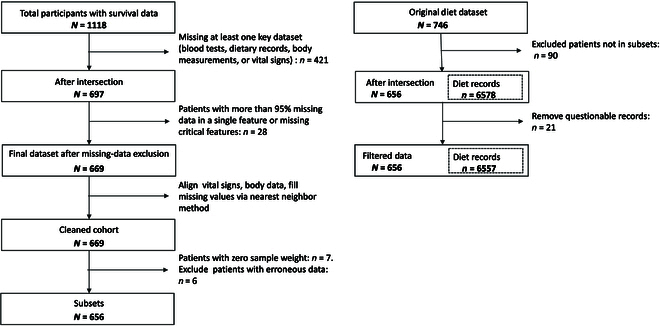
Study data selection flowchart.

A retrospective analysis of real-world data is conducted, involving 656 patients aged between 16 and 97 years, all diagnosed with ESRD. These patients were tracked at the PD clinic of Peking University Third Hospital from January 2006 to January 2018. All patients underwent long-term regular follow-ups until they are lost to follow-up, deceased, or until data collection ended on 2018 October 31. The duration of treatments varied from 2 months to 10 years. The data selection process is clearly outlined in Fig. 2.

All data are obtained from routine clinical records at the same PD clinic, encompassing baseline data at the onset of dialysis, follow-up data, and outcome data. During the data collection and processing phase, patient information is de-identified, and individual identities are linked exclusively to patient IDs. Demographic and clinical lab tests, such as age, gender, diabetes status, blood pressure, blood glucose, blood phosphorus, albumin, and more, are collected during each patient visit. The results from the initial visit served as baseline values, with their time-averaged values calculated subsequently. The specific methodology is detailed in the “Data preprocess” section. During the follow-up, all patients are required to visit the PD clinic regularly with their 3-day dietary records. These records are initially collected at the sixth month of dialysis to establish baseline data and subsequently at least every 3 months. After verification using food models by the dietitian, the dietary information from the diaries is entered into a computer software program, and dietary intake is calculated accordingly [16]. Dietary protein intake (DPI) and dietary energy intake (DEI) are normalized by ideal body weight, which is defined as body height (cm) minus 105 (modified Broca method) [17].

In our center, after PD initiation, all patients were closely followed by a dedicated nurse. All the patients were administered lactate-buffered glucose-based dialysate using a twin-bag connection system (Baxter Healthcare). Most of the patients underwent manual PD and with approximately 5% receiving automated peritoneal dialysis (APD) treatment. Patients were routinely followed until the end of PD outcomes. PD outcomes including death, renal transplantation, recovery of renal function, transferred to hemodialysis due to technical failure, transfer to other dialysis centers, or loss to follow-up were recorded routinely. The primary outcome examined in this study was all-cause mortality on PD (censored for renal transplantation, recovery of renal function, transferred to hemodialysis due to technical failure, transfer to other dialysis centers, loss to follow-up, and end of study).

#### Data preprocess

The time-averaged calculations of biochemistry and nutrients in the models are conducted based on the measurements taken over a 6-month period [15].

**Fig. 3.**
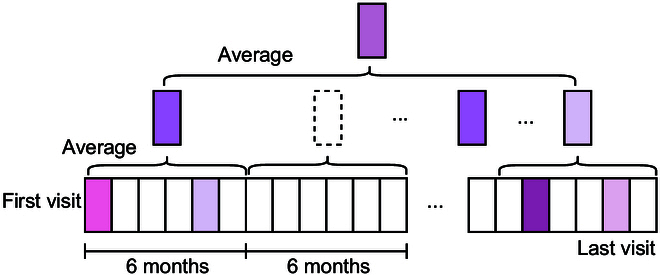
Time-averaged data processing method. The white rectangles represent months for which there are no records, while the purple rectangles indicate that there are records for the corresponding months, and different colored rectangles represent different records. The dashed boxes denote event windows where no records exist, and to ensure data accuracy, we discard such time windows. The final “time-average” data represent the time-averaged result of the original records.

The detailed methodology can be described as follows, with reference to Fig. 3. Patient follow-up records are segmented into equal-length time intervals. Within each interval, the data for all patient visits are averaged to create a single average record for that period. Depending on the varying follow-up times, there might be multiple average follow-up records available. To obtain the overall average follow-up record, the data from these records are averaged together. In cases of missing follow-up data, no imputation is performed to ensure data diversity and accuracy. When calculating the average, any corresponding records with missing data are excluded from the calculation. We set the interval to 6 months due to the irregularity of patient dietary records, which are typically documented every 3 months. Using a 6-month interval increases the likelihood of obtaining sufficient data. Furthermore, previous studies [15] on dietary research have also utilized 6-month intervals, providing a precedent for this approach. All records of patient follow-up are included to calculate the time-averaged data.

#### Data statistics

The statistical results of the data are presented in the “How do clinical characteristics vary in patients with different baseline albumin levels?” section. Continuous data are represented by mean ± SD or interquartile range, depending on the data distribution. Discrete data (categorical variables) are presented as frequencies or percentages. Group comparisons are conducted using one-way analysis of variance (ANOVA) [18,19], Tukey’s Honestly Significant Difference test [20,21], and the chi-square ($\chi^{2}$) test [22,23] for discrete data to compare differences in variables among groups. The log-rank test [24,25] is employed to evaluate differences in survival curves among the 3 groups.

The computations are performed using Python 3.7.13 and Lifelines 0.27.2, as well as R-base 4.2.2 software. All statistical tests are 2-tailed, and the threshold for statistical significance is established at *P* < 0.05. In instances where *P* < 0.01 or *P* < 0.001, special symbols denote these heightened levels of significance.

### A 2-stage model for evaluating mortality risk and modeling nonlinear nutrient-risk correlations

#### Patient subtyping

In order to achieve more detailed observations and improve the model’s applicability in clinical practice, we selected a biomarker for patient subtyping. As shown in the “How do clinical characteristics vary in patients with different baseline albumin levels?” section, our data analysis revealed significant differences in survival outcomes among individuals with varying serum albumin levels. These serum albumin levels also reflect patients’ dietary status. Other clinical findings support this choice as follows:

• **Serum albumin for prognosis prediction:** Serum albumin is an important biomarker in predicting outcome in PD patients. For example, hypoalbuminemia has been recognized as a potent predictor for PD-related peritonitis, which is a leading cause of infection-related mortality and peritonitis in patients [26]. Furthermore, some models have assigned high attention weights to albumin levels in patients who have survived, indicating that low-risk scores are correlated with higher albumin levels [27–29].
• **Serum albumin for nutritional intake evaluation:** Current studies have revealed a strong correlation between serum albumin levels and the nutritional status of patients with ESRD. Malnutrition in ESRD patients is typically associated with lower serum albumin levels, whereas higher levels usually suggest better nutritional status [4,30].
• **Availability of serum albumin in clinical practice:** Serum albumin is a routinely measured biomarker in the regular lab tests conducted during the clinical follow-up of most ESRD patients. This widespread availability ensures that our findings can be easily implemented in clinical settings, including in regions with underdeveloped healthcare infrastructure. By utilizing serum albumin as the biomarker for patient subtyping, we aim to make our research outcomes more accessible and practical.

This study serves as an initial investigative research project to confirm the relationship between dietary intake and clinical outcomes. In our future work, we plan to incorporate more comprehensive methods for patient subtyping, such as unsupervised patient clustering techniques.

#### Model stage 1: Mortality risk evaluation

To explore the range of dietary nutrient intake in different types of patients, we first assessed the associations between various dietary nutrients and patients’ mortality risk across different patient subtype groups. For the mortality risk assessment model, we chose either statistic-based models, such as the Cox proportional hazards model [31] and the logistic regression model [32]; machine learning-based models, such as the random forest model [33]; or deep learning-based models, such as neural network survival models or neural network prediction models [27,28,34,35].

In this study, we opted for the multivariate Cox proportional hazards regression model to predict and assess mortality risk. This model is widely used in the medical field due to its better stability and credibility and does not require model training. Additionally, the Cox model benefits from a time-to-event approach, which effectively handles censoring compared to other models.

The Cox proportional hazards regression model is employed to adjust for well-recognized confounding factors and determine the corresponding mortality risk associated with time-averaged dietary nutrient intake. We presented the multivariate-adjusted hazard ratios (HRs) along with their 95% confidence intervals (CIs).

#### Model stage 2: Nonlinear nutrient-risk correlation modeling

As discussed in the Introduction, extreme intake levels of certain dietary nutrients, either excessively high or low, may pose health risks to patients. This understanding has led us to investigate the nonlinear relationship between dietary nutrient intake and mortality risk.

In the initial stage, delineated in the “Model stage 1: Mortality risk evaluation” section, we established the correlation between patients’ dietary nutrient intake and mortality risk. For any specific dietary nutrient intake, we estimate the patients’ mortality risk utilizing the model. By sampling in accordance with the patients’ actual conditions, we generate several “*x*–*y*” pairs representing the relationship between dietary nutrient intake and mortality risk.

To more accurately establish the beneficial dietary nutrient intake threshold, we recognize that a nonlinear correlation may exist between dietary nutrients and mortality risk. Consequently, we further utilize the restricted cubic spline (RCS) method [36,37] to fit the results previously mentioned. In the fitted results, we identify the nutrient range $\left(r_{\min},\;r_{\max}\right)$ that corresponds to an HR value less than 1 (i.e., the corresponding intake range is a protective factor) and designate this as the suitable nutrient intake range. Subsequently, we utilize the ANOVA method to test for nonlinearity. If the *P* value indicates a significant nonlinear relationship, it implies that the intake of the dietary nutrient in question has a nonlinear association with mortality risk. This suggests that the identified range is meaningful. Conversely, if the nonlinearity is not significant, it may indicate that there is no apparent nonlinear relationship, and the range may only delineate an upper or lower limit. In such cases, the results of the linear test from stage 1 can be considered as a reference.

## Results

We conducted experiments to address the following research questions.

• RQ1. How do clinical characteristics vary in patients with different baseline albumin levels?
• RQ2. How does dietary nutrition affect the mortality rate in patients?
• RQ3. What are the beneficial dietary intake ranges for each nutrition?

### How do clinical characteristics vary in patients with different baseline albumin levels?

We found that albumin is an important indicator for distinguishing the health status of patients, as it is associated with both mortality risk and dietary management. This makes albumin a confounding variable in the study of the relationship between dietary nutrition and mortality risk.

**Fig. 4.**
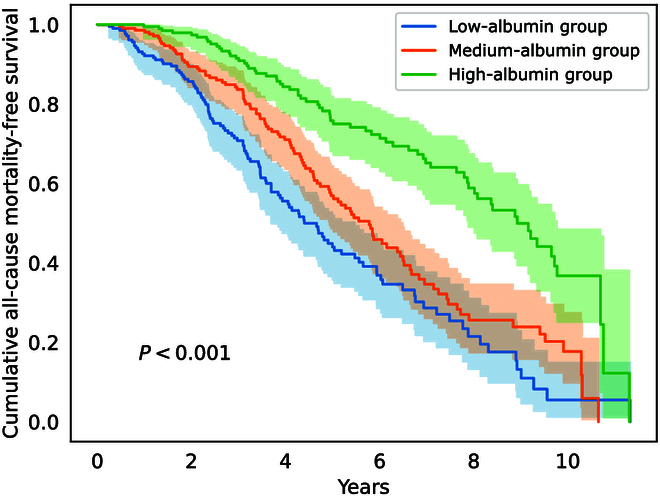
Cumulative all-cause mortality-free survival curves by albumin levels. The study includes 656 patients, with 289 endpoint events of death recorded, and a total of 2,723 person-years of data. The study subjects were divided into 3 albumin level groups: low-albumin group (albumin levels below 35.2 g/l, *n* = 217), medium-albumin group (albumin levels between 35.2 and 39.5 g/l, *n* = 222), and high-albumin group (albumin levels above 39.5 g/l, *n* = 217).

To begin with, Kaplan–Meier survival analysis showed significant differences in survival curves among patients with different baseline albumin levels (*P* < 0.001) (see Fig. 4). Additionally, we validated the differences in other clinical indicators among patients with different albumin levels, showing variations in several health-related indicators between groups (see Table 1). Furthermore, we discovered that the baseline levels of dietary nutrients did not differ significantly among patients with different albumin levels (see Table 2), indicating that patients did not pay special attention to their diet before the follow-up. However, the average dietary intake over time, a method to calculate the daily average diet during the follow-up period, showed differences (see Table 3), suggesting that patients adjusted their diet according to their health status based on medical advice during the follow-up. Therefore, albumin is correlated with both the health status and dietary habits of patients. Grouping patients by albumin levels can help reduce the impact of confounding variables to some extent and is clinically useful. Specifically, we classify them into 3 categories using tertiles [12]: low, medium, and high, based on their baseline albumin levels. The ranges for these categories are below 35.2 g/l, 35.2 to 39.5 g/l, and above 39.5 g/l, respectively, with the groups consisting of 217, 222, and 217 individuals. The distribution of baseline clinical indicators and dietary nutrient intake for these groups are presented in Tables 1 and 2.

**Table 1.** Baseline demographic characteristics and clinical laboratory findings by albumin level groups. The study includes 656 patients, with 289 endpoint events of death recorded, and a total of 2,723 person-years of data. The study subjects were divided into 3 albumin level groups: low-albumin group (albumin levels below 35.2 g/l, *n* = 217), medium-albumin group (albumin levels between 35.2 and 39.5 g/l, *n* = 222), and high-albumin group (albumin levels above 39.5 g/l, *n* = 217).

Variables	Unit	All		Low-albumin group		Medium-albumin group		High-albumin group		*P* ^a^
		Mean	SD	Mean	SD	Mean	SD	Mean	SD	
Age	year	59.11	15.78	63.39	14.03	60.27	15.85	53.64	15.84	<0.001
Gender	-	Female 327 (50%)		Female 100 (46%)		Female 117 (53%)		Female 110 (51%)		0.34
Height	cm	162.60	9.46	162.86	8.68	161.89	10.47	163.08	9.10	0.81
Weight	kg	61.17	11.95	59.95	12.13	60.44	11.58	63.13	11.95	<0.01
BMI	-	23.19	5.89	22.50	3.66	23.42	8.71	23.66	3.67	0.04
Albumin	g/l	37.15	4.90	31.58	3.00	37.56	1.20	42.29	2.12	<0.001
Diab	-	Diabetes 244 (37%)		Diabetes 121 (56%)		Diabetes 76 (34%)		Diabetes 47 (22%)		<0.001
SBP	mmHg	135.65	21.38	137.24	21.97	135.15	21.69	134.58	20.44	0.19
DBP	mmHg	78.97	14.20	75.74	14.55	79.37	14.42	81.79	12.98	<0.001
WBC	×10^9^/l	6.88	2.28	6.88	2.46	6.77	2.40	7.00	1.94	0.58
Hb	g/l	108.11	21.78	104.12	20.54	109.05	21.70	111.15	22.55	<0.001
Urea	mmol/l	21.26	6.54	20.02	6.91	21.13	5.82	22.63	6.62	<0.001
Scr	μmol/l	720.38	254.87	616.76	225.81	734.61	243.01	809.44	258.08	<0.001
K	mmol/l	4.33	0.78	4.23	0.87	4.32	0.73	4.45	0.70	<0.01
Na	mmol/l	139.17	4.87	138.51	7.06	139.13	3.66	139.88	2.71	<0.01
Cl	mmol/l	101.48	6.33	101.63	6.31	100.76	7.47	102.06	4.86	0.48
Ca	mmol/l	2.24	0.84	2.09	0.28	2.25	0.29	2.39	1.39	<0.001
P	mmol/l	1.61	0.49	1.52	0.45	1.63	0.44	1.69	0.55	<0.001
Hs-CRP	mg/l	5.66	13.29	7.45	18.04	5.73	11.19	3.78	8.69	<0.01
Glucose	mmol/l	6.19	2.94	6.74	3.88	6.09	2.13	5.74	2.45	<0.001
CO2CP	mmol/l	25.87	4.44	26.56	4.33	26.13	4.42	24.93	4.43	<0.001
GFR	ml/min	0.06	0.60	0.12	1.04	0.04	0.01	0.03	0.02	0.14

Cl, chloride; CO2CP, carbon dioxide combining power; WBC, white blood cells; Hb, hemoglobin; Ca, calcium; K, potassium; Na, sodium; Scr, serum creatinine; P, phosphorus; Hs-CRP, high-sensitivity C-reactive protein; SBP, systolic blood pressure; DBP, diastolic blood pressure; Diab, diabetes; BMI, body mass index; GFR, glomerular filtration rate

^a^ The term “*P*” represents the overall test difference. The threshold for statistical significance was established at *P* < 0.05. In instances where *P* < 0.01 or *P* < 0.001, special symbols will denote these heightened levels of significance.

**Table 2.** Baseline dietary profiles by albumin level groups. The study includes 656 patients, with 289 endpoint events of death recorded, and a total of 2,723 person-years of data. The study subjects were divided into 3 albumin level groups: low-albumin group (albumin levels below 35.2 g/l, *n* = 217), medium-albumin group (albumin levels between 35.2 and 39.5 g/l, *n* = 222), and high-albumin group (albumin levels above 39.5 g/l, *n* = 217).

Variables	Unit	All		Low-albumin group		Medium-albumin group		High-albumin group		*P* ^a^
		Mean	SD	Mean	SD	Mean	SD	Mean	SD	
Water	g/d	1,520.81	572.28	1,486.04	561.38	1,490.19	585.03	1,586.90	566.77	0.07
Protein	g/d	52.01	18.30	51.40	17.36	50.96	18.89	53.69	18.57	0.19
Fat	g/d	58.32	24.01	57.85	23.15	56.65	22.79	60.49	25.93	0.25
Carbohydrate	g/d	214.39	82.84	197.58	70.78	214.50	82.95	231.09	90.52	<0.001
Calories	kcal/d	1,544.92	523.92	1,471.84	477.13	1,528.79	518.59	1,634.50	562.02	<0.01
Df	g/d	9.89	5.90	9.18	4.37	10.04	5.86	10.44	7.10	0.03
K	mg/d	1,475.64	591.13	1,431.76	543.80	1,457.93	625.32	1,537.64	598.10	0.06
Na	mg/d	2,307.36	1,182.15	2,432.96	1,285.46	2,267.54	1,207.20	2,222.48	1,034.19	0.06
Mg	mg/d	246.99	98.91	239.63	89.11	244.99	100.59	256.40	105.96	0.08
Ca	mg/d	445.67	230.32	457.24	248.81	423.02	213.19	457.28	227.29	1.00
P	mg/d	792.38	283.65	779.30	264.41	783.71	282.33	814.33	302.94	0.20
Fe	mg/d	15.29	6.58	14.86	5.75	15.36	7.16	15.65	6.72	0.21
Zn	mg/d	7.87	3.33	7.80	3.15	7.89	3.57	7.93	3.26	0.67
Cu	mg/d	1.33	0.89	1.19	0.76	1.28	0.63	1.51	1.16	<0.001
Mn	mg/d	3.93	1.86	3.64	1.58	3.97	1.94	4.18	2.00	<0.01
Se	μg/d	36.77	21.34	35.62	16.06	35.34	15.07	39.39	29.68	0.07
Retinol	μg/d	166.30	207.21	177.98	179.76	165.28	245.23	155.67	190.00	0.26
Vitamin A	μg RAE/d	463.23	392.35	461.16	401.21	471.56	416.81	456.78	357.71	0.91
Carotene	μg/d	1,743.30	1,978.37	1,671.51	2,068.62	1,769.53	2,071.96	1,788.24	1,785.82	0.54
Vitamin E	μg/d	20.96	12.88	20.74	14.13	20.10	11.63	22.05	12.75	0.29
Thiamine	mg/d	1.66	10.08	1.98	12.96	1.16	3.71	1.86	11.22	0.90
Riboflavin	mg/d	1.73	9.85	2.11	12.86	1.22	3.38	1.88	10.80	0.81
Nicotinic	mgNE/d	11.98	6.00	11.61	5.17	12.13	6.76	12.20	5.94	0.31
Ascorbic	mg/d	88.58	72.44	83.90	71.42	85.61	68.44	96.31	76.99	0.07
DPI	g/kg/d	0.91	0.30	0.89	0.29	0.89	0.31	0.93	0.29	0.19
DEI	kcal/kg/d	26.93	8.58	25.60	8.12	26.88	8.71	28.32	8.72	<0.001

Df, dietary fiber; K, potassium; Na, sodium; Mg, magnesium; Ca, calcium; P, phosphorus; Fe, iron; Zn, zinc; Cu, copper; Mn, manganese; Se, selenium; Thiamine, vitamin B1; Riboflavin, vitamin B2; Nicotinic, nicotinic acid; Ascorbic, ascorbic acid; DPI, dietary protein intake; DEI, dietary energy intake

^a^ The term “*P*” represents the overall test difference. The threshold for statistical significance was established at *P* < 0.05. In instances where *P* < 0.01 or *P* < 0.001, special symbols will denote these heightened levels of significance.

**Table 3.** Time-average dietary profiles by albumin level groups. The study includes 656 patients, with 289 endpoint events of death recorded, and a total of 2,723 person-years of data. The study subjects were divided into 3 albumin level groups: low-albumin group (albumin levels below 35.2 g/l, *n* = 217), medium-albumin group (albumin levels between 35.2 and 39.5 g/l, *n* = 222), and high-albumin group (albumin levels above 39.5 g/l, *n* = 217).

Variables	Unit	Recommended intake^a^	All		Low-albumin group		Medium-albumin group		High-albumin group		*P* ^b^
			Mean	SD	Mean	SD	Mean	SD	Mean	SD	
Water	g/d	No clear numerical value. For patients with stable fluid status, the daily fluid intake should be 500 ml plus the previous day’s urine output and the previous day’s net peritoneal dialysis ultrafiltration amount [49].	1,512.00	522.61	1,441.07	471.74	1,518.35	495.35	1,574.88	585.45	<0.01
Protein	g/d	see DPI	52.52	15.78	49.91	13.48	52.50	14.43	55.09	18.50	<0.001
Fat	g/d	25% to 35% of total calories [50]	61.28	17.76	58.63	17.49	61.71	16.84	63.45	18.61	<0.01
Carbohydrate	g/d	The recommended carbohydrate energy contribution is between 55% and 65% [50].	215.34	68.68	192.29	54.77	216.08	64.83	237.06	77.04	<0.001
Calories	kcal/d	see DEI	1,582.30	450.26	1,451.05	374.35	1,593.96	423.44	1,698.71	507.52	<0.001
Df	g/d	19.8 g/d [51]	10.18	3.75	9.03	3.42	10.52	3.86	10.96	3.69	<0.001
K	mg/d	Normal 2,000 mg/d [52]	1,493.33	481.10	1,388.68	428.49	1,525.76	503.01	1,563.75	492.45	<0.001
Na	mg/d	<2,300 mg/d [6]	2,595.51	925.01	2,511.73	892.66	2,628.97	879.93	2,644.62	994.49	0.13
Mg	mg/d	Normal 300–330 mg/d [52]	253.39	80.37	236.79	75.59	257.28	76.78	265.78	85.72	<0.001
Ca	mg/d	800–1,000 mg/d, <2,000 mg/d [6]	437.14	168.42	447.75	184.32	441.10	163.93	422.98	155.83	0.12
P	mg/d	800–1,000 mg/d [6]	799.52	244.67	764.02	220.35	804.01	227.36	829.70	277.75	<0.01
Fe	mg/d	Normal 10–18 mg/d, <42 mg/d [52]	15.70	5.17	14.72	4.94	15.75	4.75	16.59	5.60	<0.001
Zn	mg/d	Normal 8.5–12 mg/d, <40 mg/d [52]	8.18	2.87	7.77	2.51	8.26	2.65	8.49	3.34	<0.01
Cu	mg/d	Normal 0.7–0.8 mg/d, <8 mg/d [52]	1.26	0.44	1.15	0.38	1.27	0.41	1.37	0.49	<0.001
Mn	mg/d	Normal 4.0–4.5 mg/d, <11 mg/d [52]	3.92	1.56	3.51	1.27	3.96	1.38	4.28	1.87	<0.001
Se	μg/d	Normal 60 μg/d, <400 μg/d [52]	35.71	11.36	34.44	10.95	34.96	10.25	37.68	12.50	<0.01
Retinol	μg/d	-	166.44	152.01	193.04	208.15	152.79	99.26	153.78	124.27	<0.01
Vitamin A	μg RAE/d	normal 600–770 μg RAE/d, <30 μg RAE/d [52]	469.28	272.27	473.60	314.27	469.66	248.05	464.70	251.00	0.73
Carotene	μg/d	-	1,705.64	1,228.01	1,503.12	1,238.72	1,786.06	1,211.35	1,824.82	1,214.32	<0.01
Vitamin E	μg/d	Normal <700 μg/d [52]	21.99	9.31	19.82	7.89	22.43	8.67	23.68	10.71	<0.001
Thiamine	mg/d	Normal 1.2–1.4 mg/d [52]	1.24	3.73	1.90	6.25	0.85	1.16	0.96	1.07	<0.01
Riboflavin	mg/d	Normal 1.2–1.4 mg/d [52]	1.32	3.58	2.01	6.00	0.96	1.05	1.00	0.96	<0.01
Nicotinic	mgNE/d	Normal 12–15 mg NE/d [52]	12.45	5.15	11.59	4.41	12.43	4.51	13.32	6.19	<0.001
Ascorbic	mg/d	Male 90, female 75 [6]	88.43	48.69	80.11	51.27	91.20	45.00	93.84	48.69	<0.01
DPI	g/kg/d	1.0–1.2 g/kg/d [6]	0.92	0.26	0.88	0.24	0.93	0.24	0.94	0.29	<0.01
DEI	kcal/kg/d	25–35 kcal/kg/d [6]	27.60	7.37	25.44	6.70	28.29	7.13	29.04	7.76	<0.001

Df, dietary fiber; K, potassium; Na, sodium; Mg, magnesium; Ca, calcium; P, phosphorus; Fe, iron; Zn, zinc; Cu, copper; Mn, manganese; Se, selenium; Thiamine, vitamin B1; Riboflavin, vitamin B2; Nicotinic, nicotinic acid; Ascorbic, ascorbic acid; DPI, dietary protein intake; DEI, dietary energy intake

^a^ In the “Recommended intake” column, the table lists the recommended dietary nutrition intake for patients with chronic kidney disease as suggested by existing guidelines or other single studies. In the absence of specific guidance for this patient group, the table provides the recommendations for the general population, which are denoted by “normal”.

^b^ The term “*P*” represents the overall test difference. The threshold for statistical significance was established at *P* < 0.05. In instances where *P* < 0.01 or *P* < 0.001, special symbols will denote these heightened levels of significance.

The average age of the entire sample is 59.1 years (SD = 15.8), and the average ages for the high-, medium-, and low-albumin groups are 53.6 years (SD = 15.8), 60.27 years (SD = 15.9), and 63.39 years (SD = 14.0), respectively. Among the total sample, 50% are female, and 37% had been diagnosed with diabetes.

Significant disparities are observed in most baseline clinical indicators and demographic characteristics across the different albumin groups, except for serum chloride levels, white blood cell count, systolic blood pressure, gender, height, and glomerular filtration rate (GFR). In contrast, the variations in the majority of baseline dietary nutrient intake indicators among the albumin groups did not reach statistical significance (*P* ≥ 0.05), with the notable exceptions of carbohydrates, calories, dietary fiber, copper, and manganese. Intergroup comparisons revealed marked differences in the intake of carbohydrates, calories, copper, and manganese between the high- and low-albumin groups (*P* < 0.05), as well as in copper intake between the high- and medium-albumin groups (*P* < 0.05).

Table 3 presents the time-averaged dietary nutrient intake results. The high concordance of our time-averaged results with the guidelines’ recommended values attests to the reliability and quality of our data.

When synthesizing information from Tables 1, 2, and 3, it becomes evident that under different serum albumin stratifications, the majority of baseline clinical indicators show significant differences (73%), whereas only a minority of baseline dietary nutrient intakes are significantly different (20%). In contrast, a substantial proportion (85%) of time-averaged dietary nutrient intakes exhibit significant differences.

**Fig. 5.**
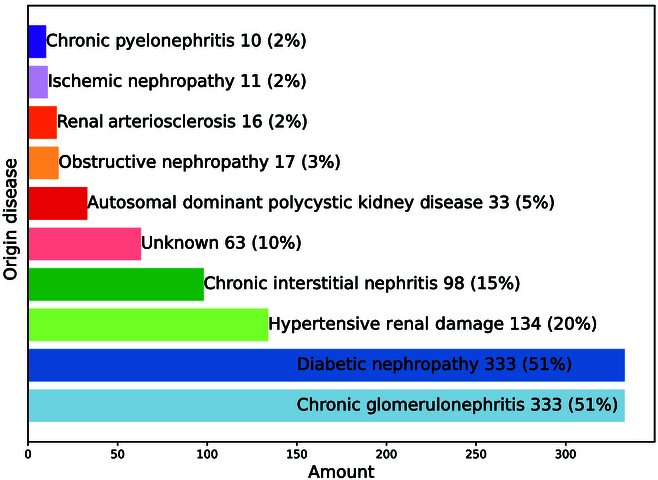
Distribution of patients’ original diseases with multiple conditions per patient possible; percentages calculated from 656 patients. The study includes 656 patients, with 289 endpoint events of death recorded, and a total of 2,723 person-years of data.

The primary disease conditions of the patients are shown in Fig. 5, with multiple conditions per patient possible. Specifically, 51% have chronic glomerulonephritis, 51% have diabetes, and 20% have hypertensive renal damage. For other conditions, please refer to Fig. 5.

### How does dietary nutrition affect the mortality rate in patients?

We performed a mortality risk association analysis for 26 commonly utilized nutrients. Table 4 presents the multivariate Cox regression analysis of mortality risk. Without considering the albumin indicator, 20 nutrients (77%) exhibit an association with mortality risk (*P* < 0.05).

**Table 4.** Evaluation of mortality risk associated with dietary nutrient intake in different albumin level groups. The study includes 656 patients, with 289 endpoint events of death recorded, and a total of 2,723 person-years of data. The study subjects were divided into 3 albumin level groups: low-albumin group (albumin levels below 35.2 g/l, *n* = 217), medium-albumin group (albumin levels between 35.2 and 39.5 g/l, *n* = 222), and high-albumin group (albumin levels above 39.5 g/l, *n* = 217).

Variables^a^	Unit	All			Low-albumin group			Medium-albumin group			High-albumin group		
		HR	95% CI	*P* ^b^	HR	95% CI	*P*	HR	95% CI	*P*	HR	95% CI	*P*
Water	100 g/d	0.95	0.92–0.99	<0.01	0.98	0.93–1.04	0.56	0.97	0.91–1.03	0.34	0.86	0.77–0.95	<0.01
Protein	g/d	0.97	0.96–0.98	<0.001	0.98	0.96–1.00	0.09	0.98	0.96–1.01	0.15	0.94	0.91–0.97	<0.001
Fat	10 g/d	0.82	0.74–0.92	<0.001	0.97	0.81–1.16	0.72	0.84	0.71–1.01	0.06	0.58	0.44–0.76	<0.001
Carbohydrate	g/d	0.99	0.99–1.00	<0.001	0.99	0.99–1.00	0.05	0.99	0.99–1.00	0.04	0.99	0.98–1.00	<0.01
Calories	10 kcal/d	0.99	0.98–0.99	<0.001	0.99	0.98–1.00	0.07	0.99	0.99–1.00	0.09	0.98	0.96–0.99	<0.001
Df	g/d	0.90	0.86–0.95	<0.001	0.93	0.86–1.00	0.06	0.92	0.85–1.00	0.06	0.80	0.70–0.90	<0.001
K	10 mg/d	0.99	0.99–1.00	<0.001	0.99	0.99–1.00	0.08	1.00	0.99–1.01	0.64	0.98	0.97–0.99	<0.001
Na	100 mg/d	0.97	0.95–0.99	<0.01	1.00	0.97–1.03	0.76	0.97	0.94–1.00	0.09	0.90	0.86–0.95	<0.001
Mg	10 mg/d	0.95	0.93–0.97	<0.001	0.97	0.93–1.01	0.12	0.98	0.94–1.02	0.36	0.87	0.81–0.93	<0.001
Ca	100 mg/d	0.89	0.81–0.98	0.02	0.96	0.84–1.11	0.58	0.90	0.77–1.05	0.18	0.79	0.61–1.01	0.06
P	10 mg/d	0.98	0.97–0.99	<0.001	0.99	0.98–1.00	0.12	0.99	0.98–1.00	0.19	0.97	0.95–0.99	<0.01
Fe	mg/d	0.94	0.91–0.98	<0.01	0.97	0.91–1.04	0.41	0.98	0.93–1.03	0.36	0.77	0.69–0.87	<0.001
Zn	mg/d	0.86	0.80–0.93	<0.001	0.91	0.81–1.02	0.10	0.97	0.87–1.09	0.66	0.61	0.49–0.76	<0.001
Cu	mg/d	0.68	0.46–0.99	0.05	0.88	0.47–1.66	0.70	0.78	0.43–1.43	0.43	0.25	0.08–0.73	0.01
Mn	mg/d	0.80	0.70–0.92	<0.01	0.89	0.74–1.07	0.20	0.98	0.78–1.23	0.85	0.41	0.26–0.64	<0.001
Se	mg/d	1.00	0.98–1.01	0.61	1.02	0.99–1.04	0.22	0.99	0.96–1.01	0.24	0.99	0.95–1.03	0.63
Retinol	10 μg/d	1.01	0.99–1.02	0.36	1.02	1.01–1.03	<0.001	0.98	0.96–1.01	0.20	0.99	0.96–1.02	0.56
Vitamin A	100 μg RAE/d	0.98	0.92–1.05	0.60	1.09	1.00–1.18	0.04	1.01	0.91–1.12	0.85	0.72	0.60–0.88	<0.001
Carotene	100 μg/d	0.99	0.98–1.01	0.45	1.00	0.98–1.02	0.77	1.02	0.99–1.04	0.21	0.91	0.87–0.96	<0.001
Vitamin E	μg/d	0.97	0.95–1.00	0.04	1.01	0.98–1.05	0.48	0.97	0.93–1.01	0.14	0.93	0.87–0.99	0.02
Thiamine	mg/d	1.02	0.99–1.04	0.18	1.02	0.99–1.05	0.23	0.99	0.85–1.14	0.85	1.22	1.01–1.47	0.04
Riboflavin	mg/d	1.02	0.99–1.05	0.17	1.02	0.99–1.05	0.22	0.97	0.82–1.15	0.76	1.24	1.02–1.50	0.03
Nicotinic	mgNE/d	0.92	0.88–0.96	<0.001	0.93	0.87–0.99	0.03	0.99	0.93–1.06	0.80	0.75	0.67–0.85	<0.001
Ascorbic	mg/d	0.99	0.99–1.00	<0.01	1.00	0.99–1.00	0.15	1.00	1.00–1.01	0.21	0.97	0.96–0.98	<0.001
DPI	0.1 g/kg/d	0.85	0.79–0.92	<0.001	0.92	0.81–1.03	0.15	0.91	0.80–1.04	0.16	0.71	0.59–0.86	<0.001
DEI	kcal/kg/d	0.94	0.92–0.97	<0.001	0.97	0.92–1.01	0.13	0.96	0.92–1.01	0.10	0.87	0.81–0.94	<0.001

Df, dietary fiber; K, potassium; Na, sodium; Mg, magnesium; Ca, calcium; P, phosphorus; Fe, iron; Zn, zinc; Cu, copper; Mn, manganese; Se, selenium; Thiamine, vitamin B1; Riboflavin, vitamin B2; Nicotinic, nicotinic acid; Ascorbic, ascorbic acid; DPI, dietary protein intake; DEI, dietary energy intake

^a^ The confounding variables we adjusted for include teh following: chloride, carbon dioxide combining power, white blood cells, hemoglobin, urea, calcium, potassium, sodium, serum creatinine, phosphorus, albumin, high-sensitivity C-reactive protein, glucose, weight, systolic blood pressure, diastolic blood pressure, diabetes, body mass index, and glomerular filtration rate. See more details at Table 1.

^b^ The term “*P*” represents the overall test difference. The threshold for statistical significance was established at *P* < 0.05. In instances where *P* < 0.01 or *P* < 0.001, special symbols will denote these heightened levels of significance.

However, this association alters when patients are classified based on their albumin levels. For the group with low albumin levels, 4 nutrients (15%) demonstrate an association with mortality risk. In the group with medium albumin levels, 3 nutrients (12%) show an association, while in the group with high albumin levels, 23 nutrients (88%) indicate an association. Specific data can be found in Table 4. This suggests that when a patient’s albumin level is high, the majority of dietary nutrients have a sensitive impact on the patient’s health status. Conversely, when a patient’s albumin level is intermediate or low, only certain key dietary nutrients are closely related to their health status.

Among the 3 patient groups, intake levels of carbohydrates, vitamin A, niacin, DPI, and DEI are all associated with an increased risk of mortality across multiple groups (*P* < 0.05). Consequently, it is imperative to carefully monitor these dietary components throughout the progression of the disease.

Moreover, in patients with high albumin levels, a significant correlation with mortality risk (*P* < 0.05) is observed for the intake of water, protein, fat, carbohydrates, calories, dietary fiber (DF), potassium (K), sodium (Na), magnesium (Mg), phosphorus (P), iron (Fe), zinc (Zn), copper (Cu), manganese (Mn), vitamin A, carotene, vitamin E, thiamine, riboflavin, niacin, ascorbic acid, DPI, and DEI. In those with medium albumin levels, carbohydrate intake, DPI, and DEI also demonstrated a significant correlation with mortality risk (*P* < 0.05). For patients presenting with low albumin levels, the intake of carbohydrates, retinol, vitamin A, and niacin is significantly correlated with mortality risk (*P* < 0.05).

**Fig. 6.**
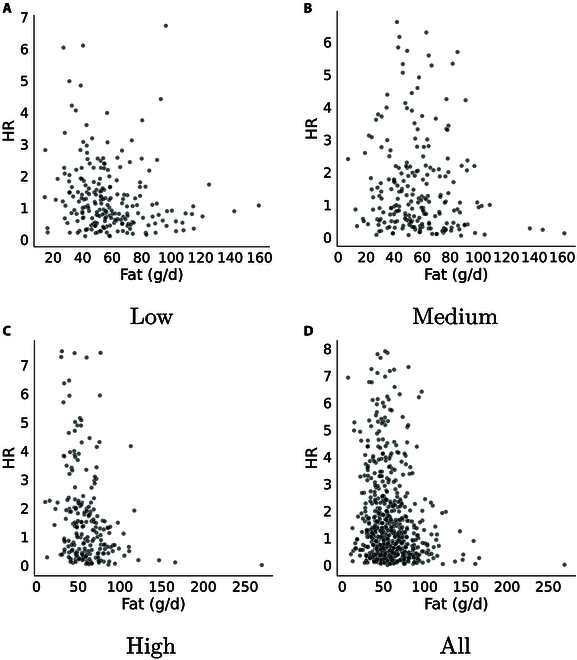
The correlation between fat intake and mortality risk among patients grouped by different albumin levels, sampled from the results of model stage 1. The study subjects were divided into 3 albumin level groups: low-albumin group (albumin levels below 35.2 g/l, *n* = 217), medium-albumin group (albumin levels between 35.2 and 39.5 g/l, *n* = 222), and high-albumin group (albumin levels above 39.5 g/l, *n* = 217). (A) to (D) respectively show the correlations for the low-albumin group, medium-albumin group, high-albumin group, and all subjects.

Figure 6 illustrates the sampling of the relationship between fat intake and mortality risk, based on the Cox model. It is evident that patients with different albumin levels display varying patterns between fat intake and mortality risk. The relationship does not definitely follow a linear pattern and warrants further investigation, despite its statistical significance in the multivariate Cox correlation analysis where it is often interpreted as linear.

### What are the beneficial dietary intake ranges for each nutrition?

The conclusions in the “How does dietary nutrition affect the mortality rate in patients?” section indicate that dietary nutrition may affect the mortality risk of PD patients. However, this is not always a simple linear relationship. In more complex scenarios, linear methods may prove inadequate, while nonlinear methods may provide more accurate results. This issue is further discussed in the “The proposed 2-stage model helps identify the nonlinear relationship” section. Table 5 presents a comprehensive overview of the specific intake ranges for various dietary nutrients that are beneficial for patients in different albumin groups (corresponds to an HR value less than 1), as determined by our 2-stage nonlinear model.

**Table 5.** Nutritional intake ranges associated with low mortality risk in patients across albumin levels. The study includes 656 patients, with 289 endpoint events of death recorded, and a total of 2,723 person-years of data. The study subjects were divided into 3 albumin level groups: low-albumin group (albumin levels below 35.2 g/l, *n* = 217), medium-albumin group (albumin levels between 35.2 and 39.5 g/l, *n* = 222), and high-albumin group (albumin levels above 39.5 g/l, *n* = 217).

Variables	Unit	All					Low-albumin group					Medium-albumin group					High-albumin group				
		A^a^	B^a^	C^a^	*P* ^b^	*P*-NL^b^	A	B	C	*P*	*P*-NL	A	B	C	*P*	*P*-NL	A	B	C	*P*	*P*-NL
Water	100 g/d	14.11	-	-	<0.01	0.10	12.76	13.72	-	0.15	0.08	13.79	-	-	0.81	0.84	14.45	-	-	0.02	0.29
Protein	g/d	50.29	74.76	-	<0.001	<0.001	52.23	62.02	-	<0.01	<0.01	46.99	48.48	-	<0.01	0.01	51.29	-	-	<0.01	0.21
Fat	10 g/d	5.91	7.72	-	<0.001	<0.001	5.88	7.36	-	<0.001	<0.001	5.78	5.84	-	<0.001	<0.01	6.04	-	-	<0.001	0.49
Carbohydrate	g/d	206.26	382.04	-	<0.001	0.02	194.82	296.37	-	<0.01	<0.001	201.57	-	-	0.22	0.81	216.48	-	-	0.02	0.63
Calories	10 kcal/d	152.47	238.33	-	<0.001	<0.001	150.10	199.18	-	<0.001	<0.001	148.13	197.21	-	0.06	0.10	156.05	-	-	<0.001	0.42
Df	g/d	9.73	15.45	-	<0.001	<0.001	9.44	14.60	-	<0.001	<0.001	9.20	9.90	13.56	0.04	0.15	10.17	16.00	-	<0.001	<0.01
K	10 mg/d	144.09	235.32	-	<0.001	<0.001	142.06	213.51	-	0.03	0.05	133.14	138.99	-	0.04	0.02	148.89	-	-	<0.001	0.07
Na	100 mg/d	24.89	24.89	-	<0.001	<0.001	21.63	24.47	-	<0.001	<0.001	20.91	24.44	-	<0.001	<0.001	25.04	-	-	<0.001	0.87
Mg	10 mg/d	24.05	40.57	-	<0.001	<0.001	23.67	36.19	-	0.02	0.01	23.95	36.13	-	0.75	0.73	24.23	-	-	<0.001	0.02
Ca	100 mg/d	4.10	7.74	-	<0.001	0.01	4.20	-	-	0.81	0.85	3.94	4.07	-	<0.01	0.01	4.01	6.61	-	0.12	0.27
P	10 mg/d	76.06	117.12	-	<0.001	<0.001	77.96	98.67	-	<0.01	<0.01	73.77	101.97	-	0.28	0.33	77.26	-	-	<0.01	0.06
Fe	mg/d	14.81	26.70	-	<0.001	<0.001	14.27	20.09	-	<0.01	<0.001	14.37	24.64	-	0.59	0.56	15.34	-	-	<0.001	0.48
Zn	mg/d	7.58	12.27	-	<0.001	<0.001	7.39	11.05	-	<0.01	<0.01	7.57	8.49	-	0.21	0.11	7.61	-	-	<0.001	0.44
Cu	mg/d	1.18	2.28	-	0.04	0.21	1.14	1.14	-	0.13	0.06	1.12	1.14	1.38	0.93	0.97	1.28	-	-	0.03	0.49
Mn	mg/d	3.64	7.24	-	<0.001	<0.01	3.53	6.27	-	0.05	0.04	2.40	3.57	5.57	0.99	0.95	3.70	-	-	<0.001	0.63
Se	μg/d	34.60	37.70	-	<0.001	<0.001	34.44	38.76	-	<0.001	<0.001	29.75	33.09	-	0.09	0.10	36.24	53.34	-	0.21	0.11
Retinol	10 μg/d	14.06	14.59	-	<0.001	<0.001	9.19	15.95	-	<0.001	0.23	11.97	12.86	-	0.02	0.02	9.92	13.00	-	0.16	0.09
Vitamin A	100 μg RAE/d	4.17	7.18	-	<0.001	<0.001	4.20	6.65	-	<0.001	<0.001	4.05	4.22	-	0.04	0.02	3.45	4.17	6.26	<0.001	0.04
Carotene	100 μg/d	14.59	32.94	-	<0.001	<0.001	13.50	36.53	-	<0.001	<0.001	14.28	21.08	-	<0.01	<0.01	15.82	-	-	<0.01	0.60
Vitamin E	μg/d	20.47	33.62	-	<0.001	<0.001	20.19	22.67	-	<0.001	<0.001	20.19	28.31	-	0.19	0.21	21.40	-	-	0.06	0.93
Thiamine	mg/d	0.71	0.71	-	<0.001	<0.001	0.70	0.72	-	<0.01	<0.01	0.56	0.63	-	0.98	0.91	0.69	-	-	0.05	0.07
Riboflavin	mg/d	-	-	-	<0.001	<0.001	0.75	0.85	-	0.11	0.18	0.77	0.78	-	0.08	0.03	0.81	1.89	-	0.06	0.10
Nicotinic	mgNE/d	11.57	20.03	-	<0.001	<0.001	11.19	19.37	-	0.02	0.02	11.28	14.54	-	<0.01	<0.01	12.19	-	-	<0.001	0.18
Ascorbic	mg/d	79.27	-	-	<0.01	0.10	75.89	-	-	0.39	0.93	78.41	79.74	-	0.06	0.11	82.74	-	-	<0.001	0.04
DPI	0.1 g/kg/d	8.75	12.83	-	<0.001	<0.001	8.73	11.18	-	<0.01	<0.01	8.49	8.61	-	0.03	0.05	8.94	-	-	<0.01	0.68
DEI	kcal/kg/d	26.22	41.93	-	<0.001	<0.01	25.86	34.28	-	<0.01	<0.001	25.69	41.98	-	0.19	0.37	26.67	27.68	28.14	<0.01	0.13

Df, dietary fiber; K, potassium; Na, sodium; Mg, magnesium; Ca, calcium; P, phosphorus; Fe, iron; Zn, zinc; Cu, copper; Mn, manganese; Se, selenium; Thiamine, vitamin B1; Riboflavin, vitamin B2; Nicotinic, nicotinic acid; Ascorbic, ascorbic acid; DPI, dietary protein intake; DEI, dietary energy intake.

^a^ Points A, B, and C define the ranges of dietary nutrition intake associated with low mortality risk, delineated as [A, B] and [C, -).

^b^ The term *P* denotes the significance of the association between nutritional elements and mortality risk, whereas *P* for nonlinear (*P*-NL) represents the statistical significance of the potential nonlinearity in the correlation between nutritional intake and mortality risk. The nonlinear relationship between nutritional elements and mortality risk is considered statistically significant only when both *P* and *P* for nonlinear are significant. It should be noted that * represents *P* < 0.05, ** signifies *P* < 0.01, and *** indicates *P* < 0.001.

**Fig. 7.**
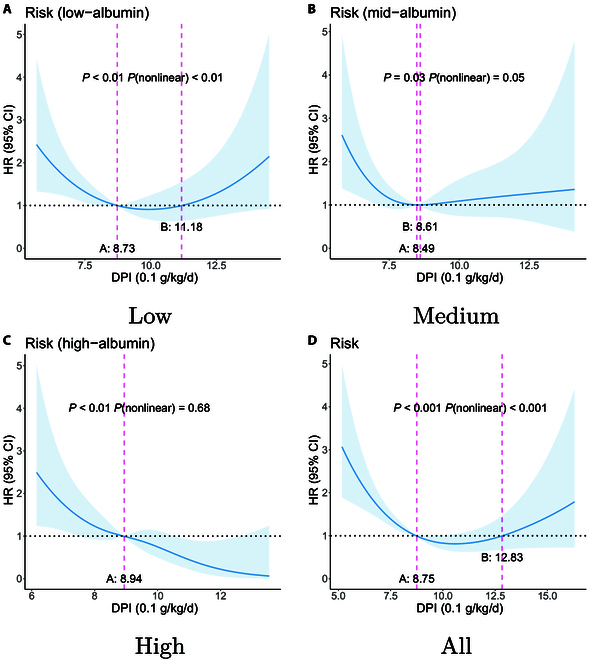
Appropriate DPI intake ranges for low mortality risk: [A, B] and [C, -). The study subjects were divided into 3 albumin level groups: low-albumin group (albumin levels below 35.2 g/l, *n* = 217), medium-albumin group (albumin levels between 35.2 and 39.5 g/l, *n* = 222), and high-albumin group (albumin levels above 39.5 g/l, *n* = 217). (A) to (D) respectively show the ranges for the low-albumin group, medium-albumin group, high-albumin group, and all subjects.

**Fig. 8.**
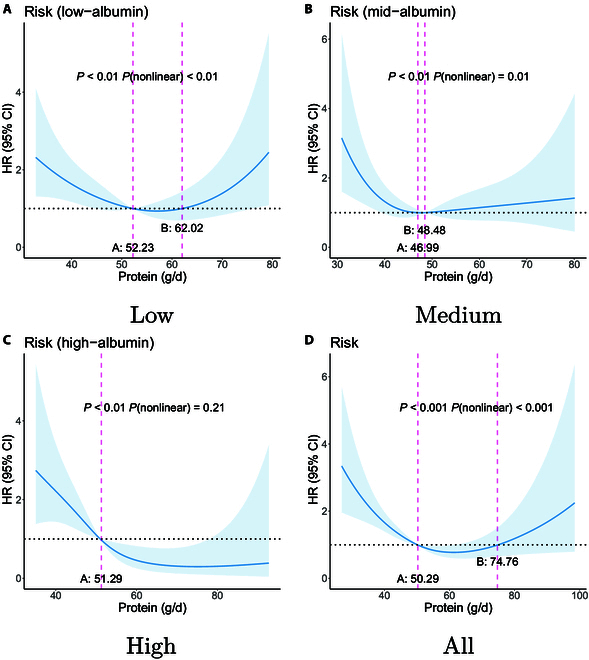
Appropriate protein intake ranges for low mortality risk: [A, B] and [C, -). The study subjects were divided into 3 albumin level groups: low-albumin group (albumin levels below 35.2 g/l, n = 217), medium-albumin group (albumin levels between 35.2 and 39.5 g/l, n = 222), and high-albumin group (albumin levels above 39.5 g/l, n = 217). (A) to (D) respectively show the ranges for the low-albumin group, medium-albumin group, high-albumin group, and all subjects.

The tables are designed to provide comprehensive insights into the dietary patterns across these groups, highlighting nutritional components such as water, protein, fat, carbohydrates, and other macronutrients, as well as micronutrients such as copper, phosphorus, and vitamin A, among others. For each nutrient, the table presents the following: (a) the unit of measurement for dietary intake, and (b) the dietary intake ranges correlated with lower mortality risk across various albumin level groups—low, medium, and high—as well as for the overall patient cohort. Here, we present the identified beneficial intake ranges associated with lower mortality risk (HR < 1) for DPI and protein in Figs. 7 and 8. Figures for all nutrients can be found in the Supplementary Materials D.

In most cases, points A and B indicate the nutrient intake range associated with low mortality risk, as illustrated in Fig. 7A and D. In some instances, point B may not exist, as shown in Fig. 7C, where the range associated with low mortality risk could be [A, -). Alternatively, there may be a point C, where the dietary nutrition intake ranges associated with low mortality risk could be [A,B] and [C, -). In our results, no scenarios involve ranges defined by more than 3 points; specific cases should be interpreted with reference to points A, B, and C, along with their corresponding *P* values.

As demonstrated in Table 5, the dietary nutrients observed to correlate with a lower risk of mortality, based on our method, are consistent with the recommended dietary nutrient intakes suggested by the guidelines.

In the group with low albumin levels, a nonlinear correlation is observed between the risk of mortality and the intake of most dietary nutrients (69%), including protein, fat, carbohydrates, calories, dietary fiber, potassium, sodium, magnesium, phosphorus, iron, zinc, selenium, vitamin A, carotene, vitamin E, thiamine, nicotinic acid, and DPI. For example, the optimal intake is determined to be 194.82 to 296.37 g/d for carbohydrates and 9.44 to 14.6 g/d for dietary fiber. This implies that for patients presenting low albumin levels, it is necessary to strictly regulate the intake of many nutrients, keeping in mind both the upper and lower limits, in order to prevent excessive or insufficient consumption.

For the group with intermediate albumin levels, only certain dietary (35%) nutrients exhibited a correlation with mortality, such as protein, fat, potassium, sodium, calcium, retinol, vitamin A, carotene, and nicotinic acid. Dietary fiber, riboflavin, and DPI are found to have a correlation with the risk of mortality, but the nonlinear test did not demonstrate statistical significance. For patients with intermediate albumin levels, only the intake of key dietary nutrients might significantly impact their health and, thus, should be regulated within the appropriate range. The intake of other dietary nutrients may not necessitate particular attention, or they may refer to the results of the “Regardless of Albumin Group” as presented in Table 5.

In the group with high albumin levels, most dietary nutrients (81%) exhibited an association with the risk of mortality, but only a subset (15%) demonstrated statistical significance in their nonlinear correlation, such as Df, Mg, vitamin A, and ascorbic acid, among others. This suggests that during periods of relatively high albumin levels in patients, the regulation of dietary nutrient intake is crucial. However, for most dietary nutrients, it may only be necessary to consider either their upper or lower limits. For example, the DPI should preferably not fall below 0.82 g/(kg·d), which is in accordance with the guidelines provided by Ikizler et al. [6].

For each dietary nutrient and its association with the risk of mortality, we can generate 4 figures, such as Figs. 7 and 8. The figures provide a visual representation of the dietary nutrient ranges correlated with reduced mortality risk, as detailed in Table 5, and include the 95% CIs.

Specifically, Fig. 8 delineates the influence of protein intake on the mortality risk of patients across different protein groups. In all protein groups, total protein intake exhibits a significant association with mortality risk (*P* < 0.05). Notably, the correlation between protein intake and mortality risk in the low-protein and medium-protein groups is nonlinear. The protein intake range associated with low mortality risk for the low-protein group is 52.23 to 60.02 g/d, while for the medium-protein group, it is 46.99 to 48.48 g/d. Conversely, the association between protein intake and mortality risk in the high-protein group is linear, with an HR of 0.94 (95% CI 0.91, 0.97), *P* < 0.001. Without distinguishing between protein groups, the suitable intake range is 50.29 to 74.76 g/d.

Figure 7 illustrates the impact of DPI intake on the mortality risk of patients across different protein groups. In all protein groups, DPI intake shows a significant correlation with mortality risk (*P* < 0.05). The relationship between DPI intake and mortality risk in the low-protein group is nonlinear, with the recommended intake range being 0.87 to 1.11 g/kg/d.

## Discussion

### The value of long-term, expert-supervised dietary nutrition data

Investigations into the dietary nutrition of PD patients within the ESRD demographic have been notably limited. This scarcity of research is, in part, attributable to the prolonged disease progression characteristic of PD, which necessitates an extended period for data collection [38]. Moreover, the process of acquiring high-quality dietary data, under the supervision of experts, is inherently complex, requiring active participation from patients in record-keeping, as well as precise and targeted documentation by dietitians [39]. The dataset utilized in this study consists of high-quality, fine-grained longitudinal data, meticulously collected under expert supervision, and is both comprehensive and detailed, encompassing 12 years of clinical indicators and dietary logs, with follow-ups conducted at least quarterly. These data have been processed with utmost care, providing a critical foundation for subsequent analysis and experimentation.

### The proposed 2-stage model helps identify the nonlinear relationship

The methodology of the proposed 2-stage model displays substantial practicality. While the majority of existing studies have primarily established correlations between dietary nutrition and mortality risk, they have often neglected nonlinear associations [7,15]. In instances where nonlinear relationships are present, as reported in previous studies [4,7,40], linear methods such as the Cox model may become ineffective and produce outcomes that are inconsistent with the actual dynamics observed. However, our approach is capable of capturing these nonlinear associations fully and providing sound conclusions.

For example, in Table 4, the Cox analysis results show a significant correlation between DPI and mortality risk across the entire population. However, in subtypes, only the high-albumin group shows a significant correlation between DPI and mortality risk, while the low- and medium-albumin groups do not exhibit a significant association between DPI intake and mortality risk. This is inconsistent with clinical practices in that patients with low albumin levels are typically advised to more stringently control their protein intake. The failure of the linear method may likely stem from the presence of nonlinear correlations that it cannot adequately capture. Our 2-stage method, on the other hand, is designed to fully capture such nonlinear relationships.

As demonstrated in Fig. 7, our method reveals that, for both the low-albumin group (*P* < 0.01, *P*-nonlinear <0.01) and the medium-albumin group (*P* = 0.03, *P*-nonlinear = 0.05), there exists a significant and nonlinear relationship between DPI and mortality risk. For the low-albumin group, our findings suggest that a protein intake of 0.87 to 1.12 g/kg/d is beneficial for patient prognosis. For the medium-albumin group, an intake of 0.85 to 0.86 g/kg/d is associated with better outcomes. In the high-albumin group, our approach indicates a significant relationship between DPI and mortality risk (*P* < 0.01); however, the nonlinearity is not significant (*P*-nonlinear = 0.68), suggesting that an intake of <0.89 g/kg/d is associated with lower mortality risk, which aligns with the conclusions drawn from linear analyses.

Overall, our method introduces the exploration of nonlinear relationships between variables, which, compared to solely using linear methods, yields more robust and precise conclusions.

### Key findings regarding the dietary nutritional intake range associated with a reduced mortality risk

Leveraging comprehensive and precise data, combined with a 2-stage model methodology, our research investigates a broad spectrum of dietary nutrients pertinent to patients with ESRD. The key findings yielded by our approach are summarized below.

• **Protein or DPI.** Protein intake is a critical clinical metric, and it is generally recommended that patients with PD consume 1.0 to 1.2 g/kg/d of protein [6]. Our experimental results indicate that for PD patients, a protein intake ranging from 0.87 to 1.28 g/kg/d is associated with a reduced risk of mortality, which is consistent with the guidelines. Moreover, extending beyond the guidelines, we observed that excessive protein intake may pose risks for patients with hypoalbuminemia, as illustrated in Fig. 7A. However, for patients with medium or high serum albumin levels, increased protein consumption does not correlate with heightened risk, as shown in Fig. 7B and C.
• **Calories or DEI.** Clinical guidelines generally recommend a caloric intake of 25 to 35 kcal/kg/d for patients with ESRD [6]. Our research findings suggest that for patients with hypoalbuminemia, an energy intake of 26 to 42 kcal/kg/d is associated with a lower risk of mortality. In contrast, for those in the medium- and high-serum-albumin groups, an intake of over 27 kcal/kg/d correlates with a reduced mortality risk, with no clearly identified upper limit of energy intake. ESRD patients often face risks of malnutrition due to factors such as appetite loss, toxin accumulation, chronic inflammation, and socioeconomic factors. It is also noted that patients on dialysis may spontaneously decrease their protein and calorie consumption owing to uremic toxins, increases in leptin and other cytokines, and delayed gastric emptying [4], underscoring the importance of ensuring a minimum level of energy intake. Furthermore, some ESRD patients may also have comorbidities such as diabetes or cardiovascular diseases, necessitating blood sugar control and reduced cardiovascular risk, which might influence the upper limits of caloric intake.
• **Fat.** Regarding the specific intake of fats and carbohydrates, the guidelines do not stipulate exact amounts but rather recommend proportions of these macronutrients in relation to total caloric intake, as detailed in Table 3. Our experimental findings demonstrate a significant nonlinear relationship between both fat and carbohydrate intake and mortality risk in patients with CKD. An intake of 59 to 77 g/d of fats and 206 to 382 g/d of carbohydrates is associated with a lower mortality risk. More granular results can be found in Table 5. Limiting the minimum intake of fats is necessary because fats are essential components of a healthy diet, providing energy, aiding in the regulation of cholesterol and blood pressure, and facilitating the absorption of vitamins. However, excessive consumption of fat, particularly harmful types, can lead to accumulation in the blood vessels, heart, and kidneys. Saturated and trans fats, in particular, can elevate cholesterol levels and contribute to vascular occlusion. Therefore, it is also necessary to limit the maximum intake of fats [41].
• **Sodium and water.** Sodium is a mineral present in salt that plays a crucial role in regulating body fluid balance. Excessive sodium intake can lead to fluid retention, which may result in high blood pressure and swelling. Such retention places additional strain on the kidneys and heart. In clinical practice, it is generally recommended that a patient’s fluid intake is guided by the urine output and dialysis fluid losses from the previous day, as detailed in Table 3, while sodium (Na) intake should typically be less than 2,300 mg/d [6]. Our study refines this recommendation. For the low-albumin group, an intake of 2,163 to 2,447 mg/d should be a protective factor for patients. For the medium-albumin group, the range is 2,091 to 2,444 mg/d. For the high-albumin group, there may not be a need for strict limitations. Overall, when not considering albumin levels, an intake of around 2,489 mg/d is suggested. The Cox linear results from Table 4 indicate that fluid intake is a protective factor for patient prognosis. However, the nonlinear results from our proposed method suggest no significant association between fluid intake and patient prognosis (see Table 5). Given the limited capacity of dialysis to remove fluid, excessive water and salt intake increases the risk of volume overload and cardiovascular disease in patients. Therefore, it is of greater importance in clinical practice to guide patients to limit their water and salt intake to maintain volume balance.
• **Vitamin A.** Our research has uncovered a nonlinear relationship between vitamin A and mortality risk, identifying an intake range of 417 to 718 μg RAE (retinol activity equivalent)/d associated with lower mortality risk, a topic not extensively discussed in prior studies [42]. However, there is literature that indeed highlights the importance of vitamin A for dialysis patients. For instance, a case study reported a Japanese male hemodialysis patient who was diagnosed with vitamin A deficiency (VAD), emphasizing the potential risk of VAD in dialysis patients, particularly when dietary intake is insufficient [43]. Furthermore, during the process of PD, patients’ vitamin A levels may gradually increase. Despite significant peritoneal transfer and losses into the dialysis fluid, levels of vitamin A and its protein carriers remained elevated throughout the study period [44,45]. It is crucial to note that excessive levels of vitamin A in the body can lead to toxicity [46].

For other findings, please refer to Appendix C.

### Limitations and future works

While our work, leveraging reliable long-term data and a 2-stage nonlinear model design, has resulted in precise dietary intake thresholds that are protective factors for patient prognosis, it remains imperfect in terms of data collection and experimentation.

**Fig. 9.**
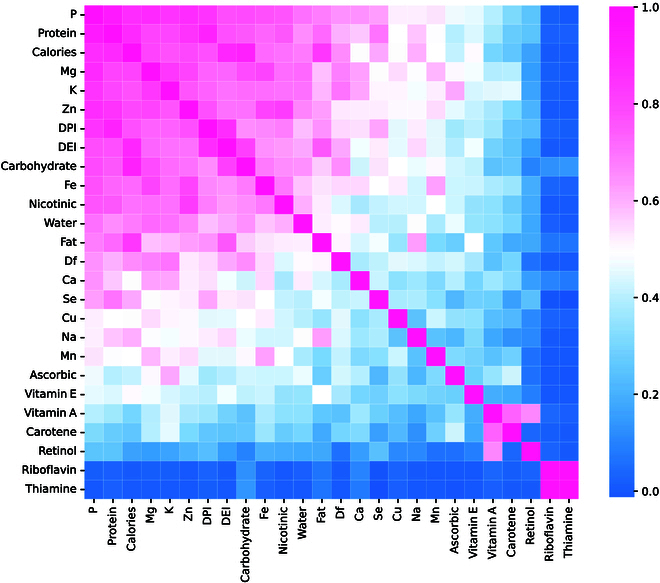
Correlation of daily dietary nutrient intake among patients with Pearson coefficients. Values close to one indicate strong correlation, and values close to zero indicate weak correlation.

• Regarding the data, our dataset does not include information on factors such as medication use or nutritional supplements. For instance, while some patients were prescribed folic acid, there are no related records available. Similarly, although some patients used nutritional supplements, these were not systematically monitored. All patients in our cohort are undergoing 24-h continuous ambulatory peritoneal dialysis. The impact of dialysate on caloric intake averages an additional 5 kcal/d based on patients’ regular intake, depending on their membrane function and glucose concentration. Previous studies have shown that this variable does not significantly affect prognosis.
• Additionally, the daily activity levels of patients, significantly associated with dietary nutrition intake and planning [47,48], are not included in our data.
• Regarding the dietary recording method, we employed the 3-day dietary record method, which, despite being one of the most accurate methods available and recommended by guidelines, requires significant effort and time from both patients and professional nutritionists. However, inaccuracies in recording are still inevitable. We look forward to the development of more convenient, accurate, and automated dietary recording solutions in the future.
• Our cohort also has its limitations. It only includes data from single-country individuals and a single center, and its applicability to other societies requires further validation. However, our framework is universal and can be applied to other cohorts. We eagerly anticipate expanding and validating our conclusions in other studies.
• There is a portion of missing data in our dataset. For dietary data, there were no missing values for individual dietary features; however, some dietary follow-up records were missing, leading to an overall missing rate of 20%. For visit data, in our study, all patients had a total of 13,239 follow-up records. The missing rates for various features were as follows: blood chloride (6%), blood carbon dioxide combining power (7%), white blood cell count (9%), hemoglobin (9%), urea (10%), calcium (11%), potassium (11%), blood sodium (12%), serum creatinine (10%), phosphorus (12%), albumin (22%), hypersensitive C-reactive protein (29%), and blood glucose (28%). We selected the most recent follow-up records of patients to fill in the missing values. The presence of missing data may affect statistical measures such as mean and variance and could potentially impact the accuracy of the results.
• In terms of experimental design, this study categorizes patients based on albumin levels, given their significant role in dietary nutrition planning. However, there is a lack of related research in this field. The complex interplay between a patient’s dietary nutrition planning and their basal metabolism, underlying diseases, disease progression stages, and various examination indicators necessitates further research and a more personalized approach. A Bayesian or causal inference-based personalized dietary nutrition recommendation could be highly beneficial. Thus, we aim to explore more advanced, user-friendly, and personalized dietary nutrition recommendation methods in our future work.
• Finally, despite the richness of our conclusions, focusing on a broader range of nutrients may complicate clinical application. To ensure our findings can be better applied in practice, we explored the correlations between dietary nutrients to identify the most critical elements for patient dietary planning (see Fig. 9), thereby providing a foundation for the practical application of our theories. In the future, we also plan to develop software that considers patients’ preferred foods and locally available ingredients, offering a simpler and more convenient dietary management solution for patients.

## Conclusions

This study advances our understanding of dietary nutrient intake and its association with mortality risk among patients undergoing PD. Leveraging a comprehensive, long-term dataset and a 2-stage analytical model, it clarifies the relationship between 26 dietary nutrients and mortality risk in PD patients, highlighting nonlinear associations for certain nutrients. The specific intake thresholds identified in this research are associated with low mortality risk and can inform effective dietary planning and management for this patient population. Furthermore, by associating personalized dietary intake ranges with albumin subtypes, this study not only strengthens existing knowledge but also establishes a groundwork for more tailored and individualized dietary approaches for PD patients. Future research will seek to include additional variables, such as patient medications and daily activity levels, to refine these associations. Additionally, it may investigate individualized dietary nutrition strategies using methods like causal inference or Bayesian models. These endeavors aim to provide more precise, actionable, and personalized nutritional guidance for patients.

## Data Availability

For further interest in the complete data for this study, please contact malt@pku.edu.cn.

## Supplementary Materials

Supplementary 1 Appendix A to E Figs. S1 to S25 Tables S1 to S3 References [53–61]
