# Supplementary material for: Exploring the Relationship between Dietary Intake and Clinical Outcomes in Peritoneal Dialysis Patients Stratified by Serum Albumin Levels: A 12-Year Follow-Up Using Fine-Grained Electronic Medical Records Data
Source: Health Data Sci. 2025 Jul 2;5:0280. doi: 10.34133/hds.0280 (PMC13182931; doi:10.34133/hds.0280)
Supplement: Supplementary 1 — Appendix A to E Figs. S1 to S25 Tables S1 to S3 References [53–61] [file hds.0280.f1.pdf]

A Appendix: Frequency distribution of patient follow-up duration.

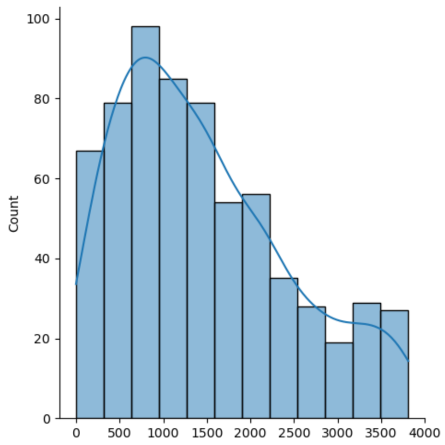

Figure S1: Frequency distribution of patient follow-up duration. The x-axis represents the follow-up duration (in days), while the y-axis represents the corresponding number of patients.

B Appendix: Comparison of existing dietary and nutritional research for ESRD patients

Table S1: Comparison of existing dietary and nutritional research for ESRD patients

	Research Question		Data Quality				Method Capability	Result Precision
Author/Year	Patient Cohort	Ending-Dependent	Long-term Follow-up Dietary Data Records Supervised by Professional Experts	Dietary Record Follow-up Duration	Dietary Record Follow-up Frequency	Nutrition Granularity	Non-linear Relationship	Precise Threshold
Metzger 2018 [7]	CKD	✗	✗	Once	Once	Fine-grained (Single Nutrition)	✓	✗
Banerjee 2019 [8]	CKD	✗	✗	Once	Once	Coarse-grained	✗	✗
Gutierrez 2014 [10]	CKD	✗	✗	Once	Once	Coarse-grained	✗	✗
Banerjee 2015 [11]	CKD	✗	✗	Once	Once	Coarse-grained	✗	✗
Wanger 2022 [9]	CKD	✗	✗	Once	Once	Fine-grained (Single Nutrition)	✗	✗
Vjojanawat 2021 [14]	PD	✗	✗	Once	Once	Fine-grained (Single Nutrition)	✗	✗
Wang 2019 [12]	PD	✗	✗	Once	Once	Fine-grained (Single Nutrition)	✗	✗
Xu 2019 [15]	PD	✓	✓	3-year	Every 3 months	Fine-grained (Single Nutrition)	✓	✗
Ours	PD	✓	✓	12-year	Every 3 months	Fine-grained (Multiple Nutrients)	✓	✓

Abbreviations: CKD: Chronic Kidney Disease;PD: Peritoneal Dialysis

C Appendix: Other findings regarding the dietary nutritional intake range associated with a reduced mortality risk

- **Phosphorus.** Elevated blood phosphorus concentrations are associated with increased mortality in patients with ESRD and contribute to a heightened risk of cardiovascular events,

primarily due to their role in vascular calcification [53]. Excessive phosphorus can also lead to secondary hyperparathyroidism, which stimulates the release of calcium from the bone matrix and results in osteodystrophy [53, 54]. Clinically, it is generally recommended that ESRD patients limit their phosphorus intake to 800-1000 mg per day. Our experimental findings are largely in agreement with these guidelines.

- **Dietary Fiber.** Moreover, it is important to highlight that the dietary fiber intake range associated with low mortality risk. Irrespective of albumin levels, we have identified a beneficial range of dietary fiber intake between 9.73 and 15.45 g/d. When albumin levels are factored into the analysis, the data suggest tailored intake ranges: 9.44-14.6 g/d for individuals with lower albumin and 10.17-16.6 g/d for those with higher albumin levels. These intake ranges correspond with, and in some cases extend, the fiber intake levels previously suggested for hemodialysis patients, which are between 10.2 and 12.4 g/d[55, 56]. Considering the proposed fiber intake for chronic kidney disease patients not on hemodialysis is as high as 19.8 g/d[40], our findings support a more generous upper limit. Furthermore, dietary fiber is acknowledged for its capacity to attenuate the glycemic impact of carbohydrate absorption[57, 58], selectively foster beneficial gut microbiota, facilitate the elimination of toxins, and improve lipid profiles, conferring renal advantages[59, 60]. Therefore, advocating for an intake range marginally above that recommended for hemodialysis patients appears substantiated. Interestingly, previous literature has documented that actual cellulose consumption by patients often does not meet these suggested levels[15], a finding that is in line with the patterns we have observed, as depicted in Table 2. This discrepancy suggests that prevalent dietary habits may fall short of supporting optimal health. Nonetheless, our two-stage method successfully delineates appropriate dietary fiber intake ranges, underscoring the robustness and utility of our investigative framework.

- **Calcium.** In the Table 5, we observed that the nutritional intake range of calcium associated with low mortality risk is lower than the recommendations provided by the guidelines. This can be attributed to the fact that for PD patients, non-dietary sources of calcium, such as calcium-phosphate binders, are often used in clinical practice. Therefore, it is normal for the dietary intake of calcium to be below the guideline recommendations for this patient group.

- **Trace Elements.** Trace elements are essential for patients with ESRD. Those undergoing dialysis frequently experience deficiencies in vitamin C, folic acid, vitamin D, iron, and zinc, and may also exhibit insufficient selenium levels, which can lead to a state of antioxidant deficiency [61]. While guidelines do not stipulate specific intake ranges for these micronutrients, our study has identified a correlation between these nutritional elements and mortality risk, as detailed in Table 5.

D Appendix: Ranges of nutrient intake associated with low mortality risk across varying albumin levels

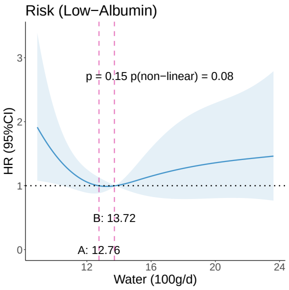

(a) Low

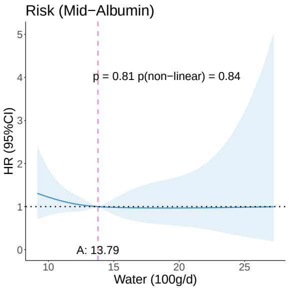

(b) Medium

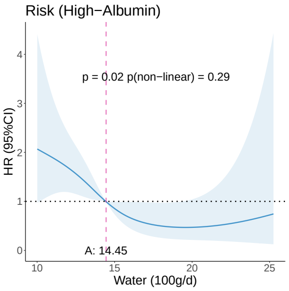

(c) High

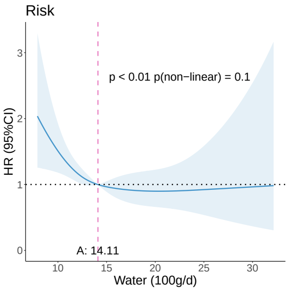

(d) All

Figure S2: Appropriate water intake ranges for low mortality risk: [A, B] and [C, -)

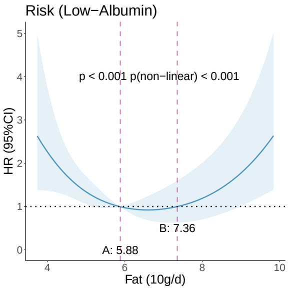

(a) Low

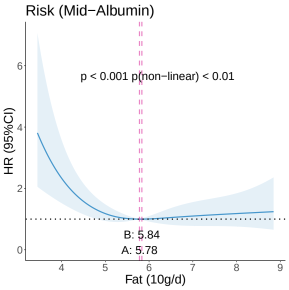

(b) Medium

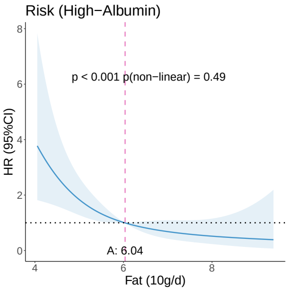

(c) High

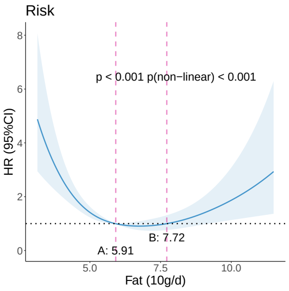

(d) All

Figure S3: Appropriate fat intake ranges for low mortality risk: [A, B] and [C, -)

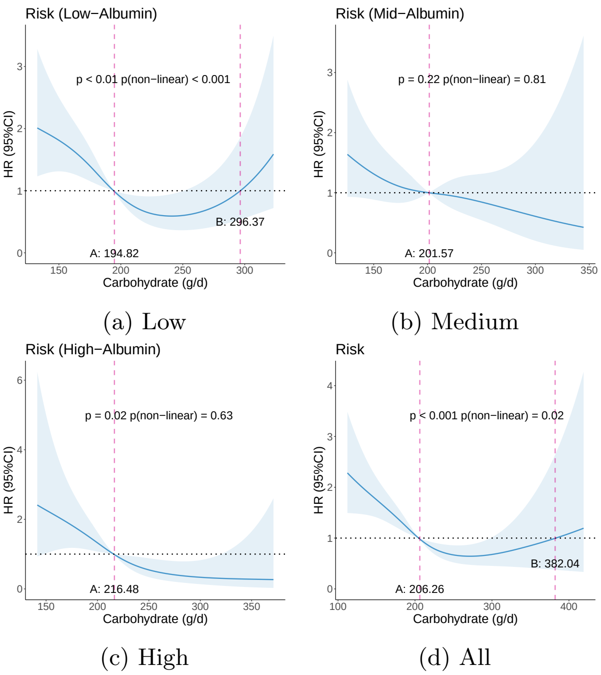

(a) Low (b) Medium

(c) High (d) All

Figure S4: Appropriate carbohydrate intake ranges for low mortality risk: [A, B] and [C, -)

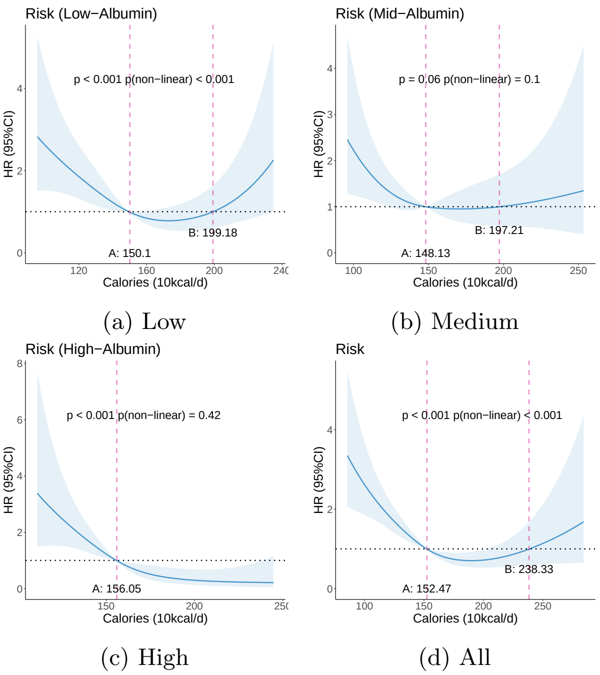

(a) Low (b) Medium

(c) High (d) All

Figure S5: Appropriate calories intake ranges for low mortality risk: [A, B] and [C, -)

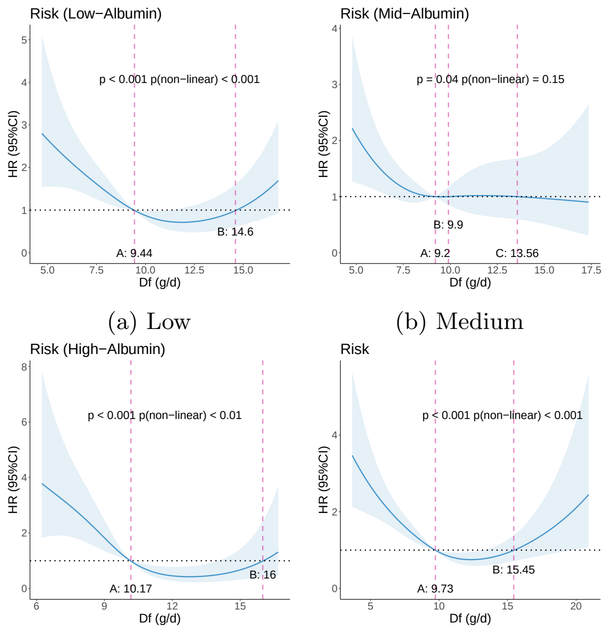

(a) Low (b) Medium

(c) High (d) All

Figure S6: Appropriate df intake ranges for low mortality risk: [A, B] and [C, -)

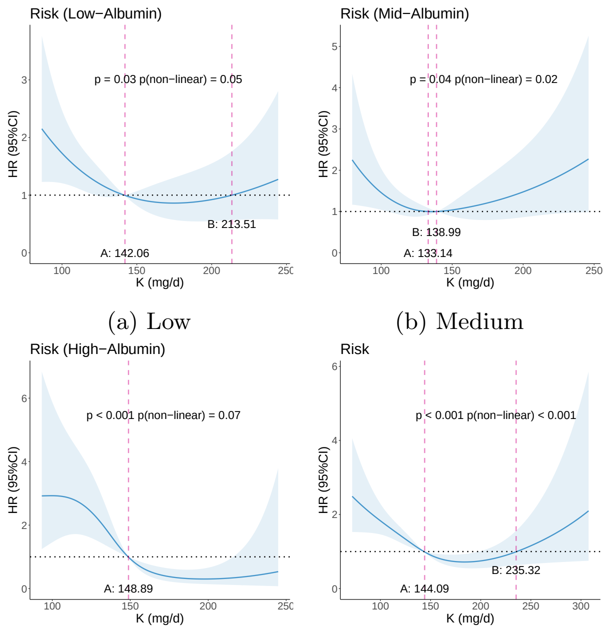

(a) Low (b) Medium

(c) High (d) All

Figure S7: Appropriate K intake ranges for low mortality risk: [A, B] and [C, -)

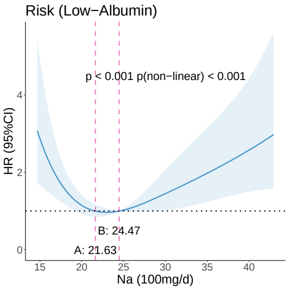

(a) Low

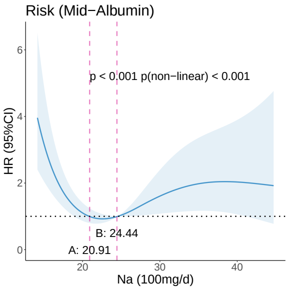

(b) Medium

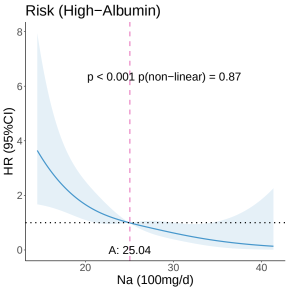

(c) High

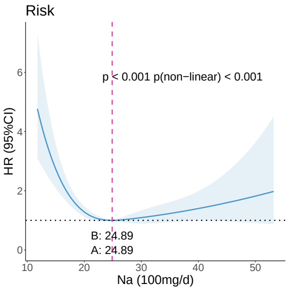

(d) All

Figure S8: Appropriate Na intake ranges for low mortality risk: [A, B] and [C, -)

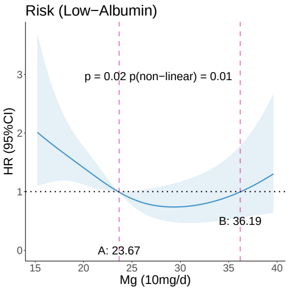

(a) Low

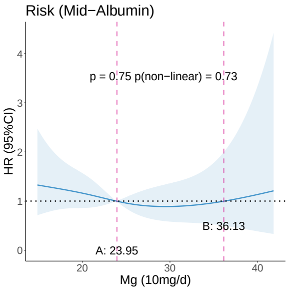

(b) Medium

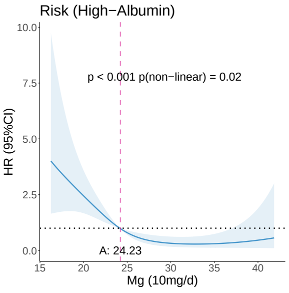

(c) High

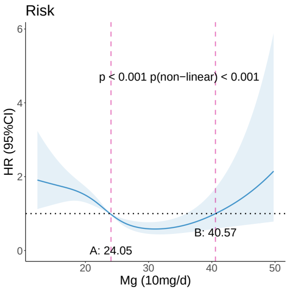

(d) All

Figure S9: Appropriate Mg intake ranges for low mortality risk: [A, B] and [C, -)

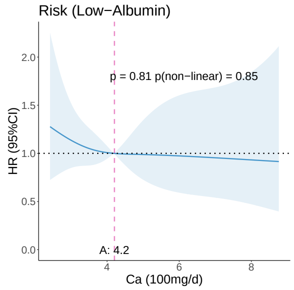

(a) Low

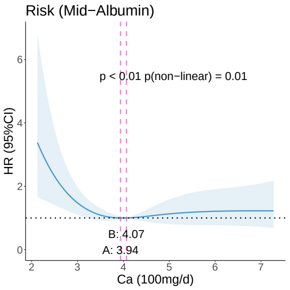

(b) Medium

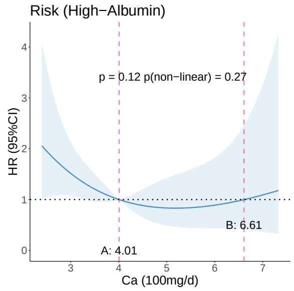

(c) High

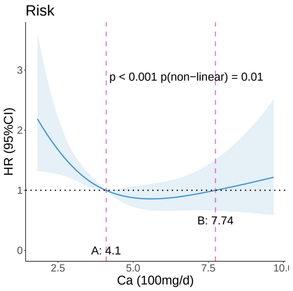

(d) All

Figure S10: Appropriate Ca intake ranges for low mortality risk: [A, B] and [C, -)

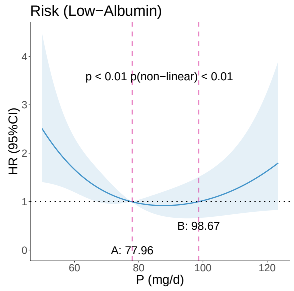

(a) Low

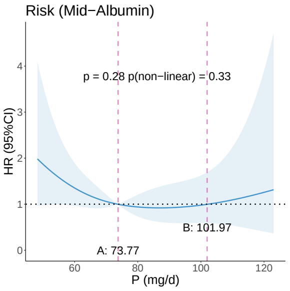

(b) Medium

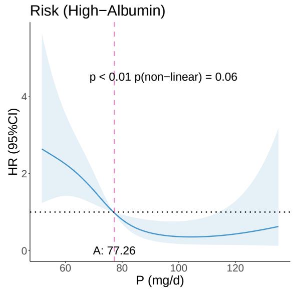

(c) High

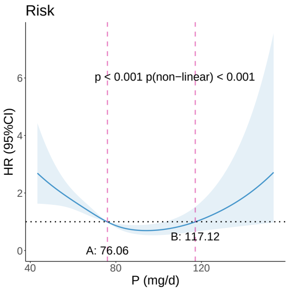

(d) All

Figure S11: Appropriate P intake ranges for low mortality risk: [A, B] and [C, -)

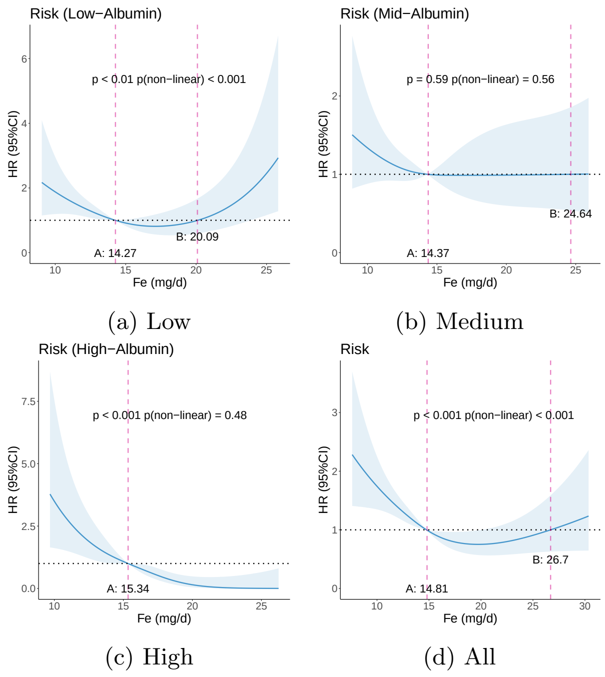

(a) Low (b) Medium

(c) High (d) All

Figure S12: Appropriate Fe intake ranges for low mortality risk: [A, B] and [C, -)

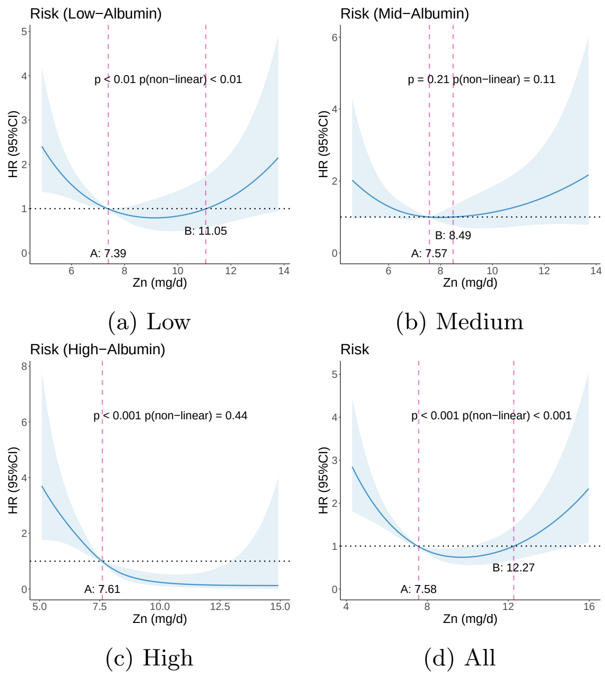

(a) Low (b) Medium

(c) High (d) All

Figure S13: Appropriate Zn intake ranges for low mortality risk: [A, B] and [C, -)

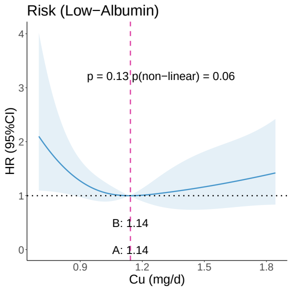

(a) Low

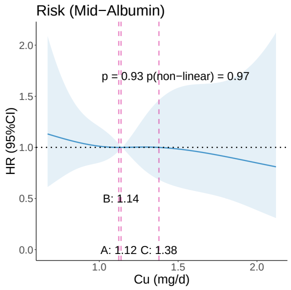

(b) Medium

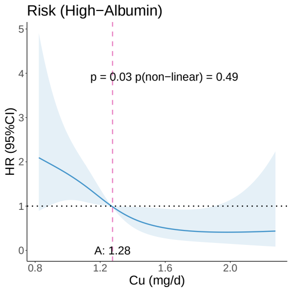

(c) High

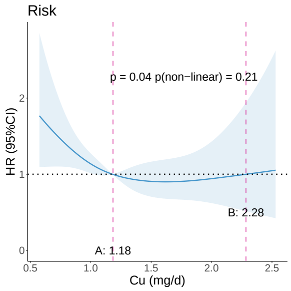

(d) All

Figure S14: Appropriate Cu intake ranges for low mortality risk: [A, B] and [C, -)

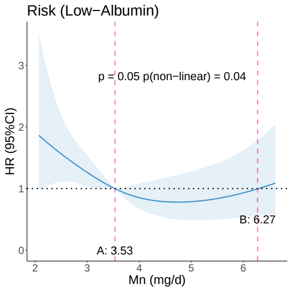

(a) Low

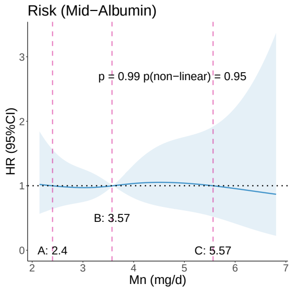

(b) Medium

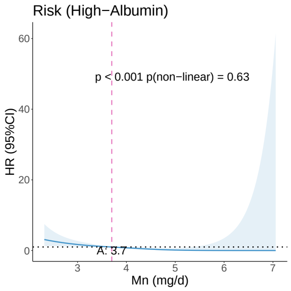

(c) High

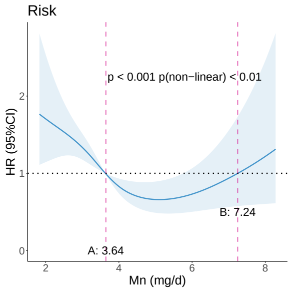

(d) All

Figure S15: Appropriate Mn intake ranges for low mortality risk: [A, B] and [C, -)

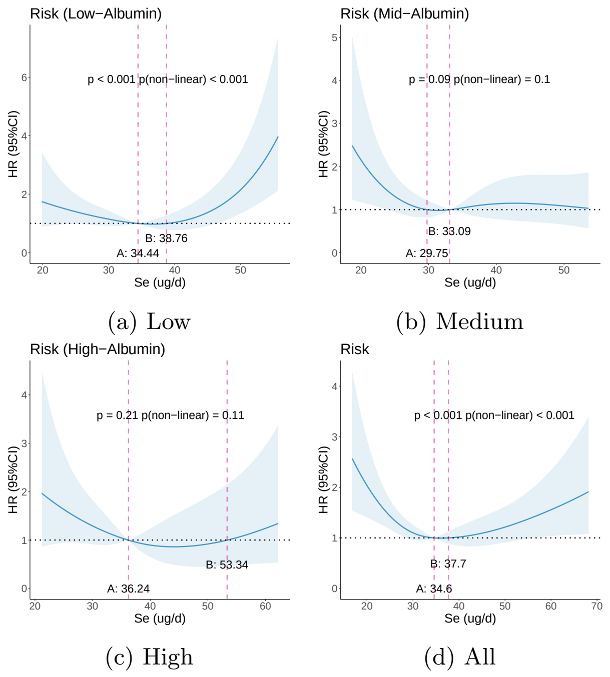

(a) Low (b) Medium

(c) High (d) All

Figure S16: Appropriate Se intake ranges for low mortality risk: [A, B] and [C, -)

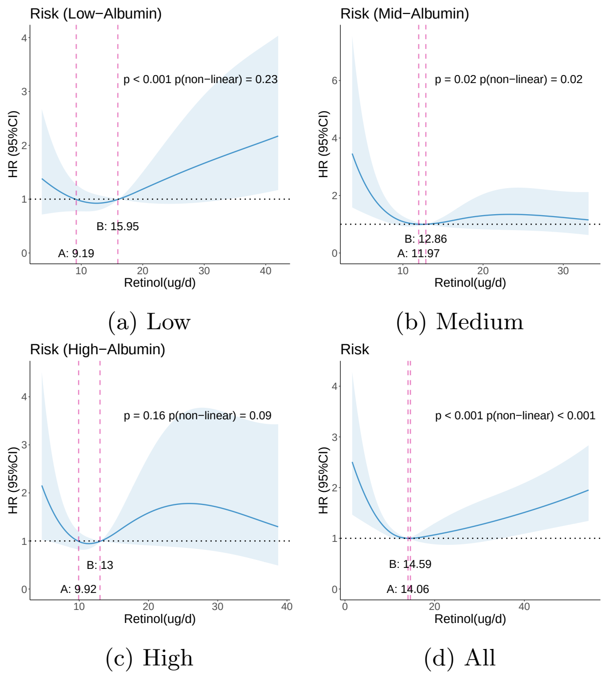

(a) Low (b) Medium

(c) High (d) All

Figure S17: Appropriate retinol intake ranges for low mortality risk: [A, B] and [C, -)

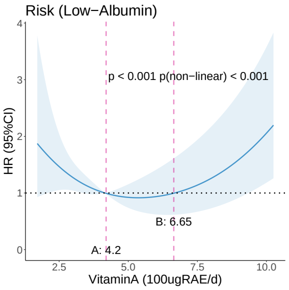

(a) Low

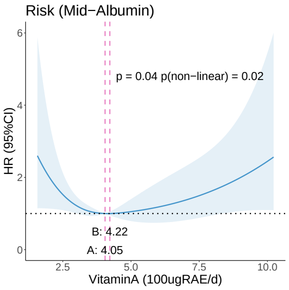

(b) Medium

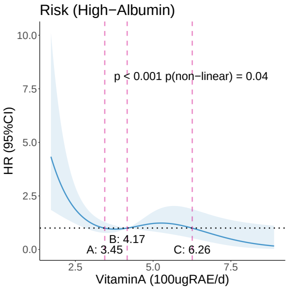

(c) High

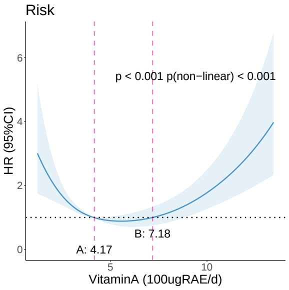

(d) All

Figure S18: Appropriate vitaminA intake ranges for low mortality risk: [A, B] and [C, -)

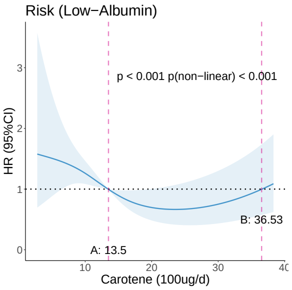

(a) Low

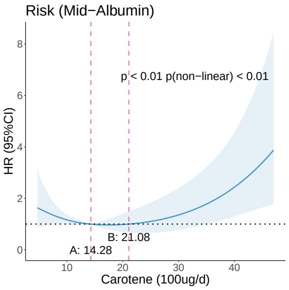

(b) Medium

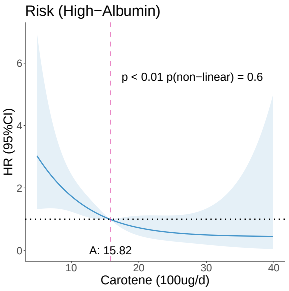

(c) High

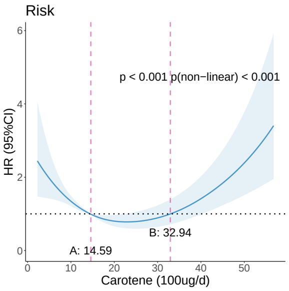

(d) All

Figure S19: Appropriate carotene intake ranges for low mortality risk: [A, B] and [C, -)

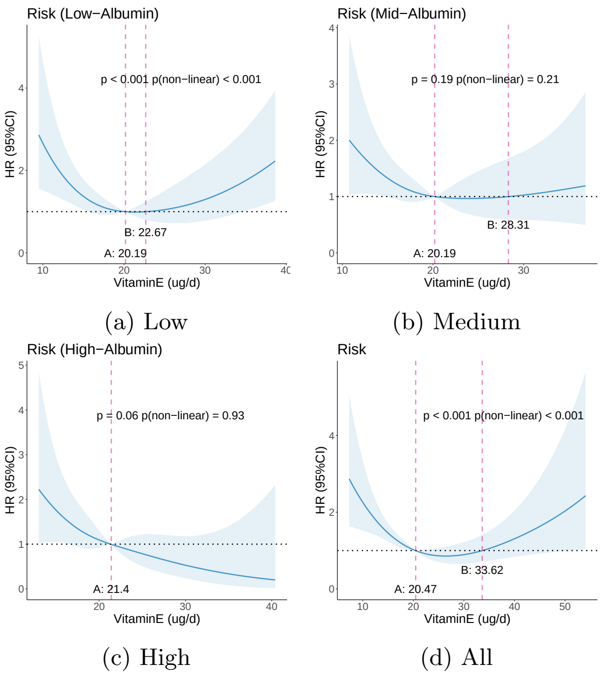

(a) Low (b) Medium

(c) High (d) All

Figure S20: Appropriate vitaminE intake ranges for low mortality risk: [A, B] and [C, -)

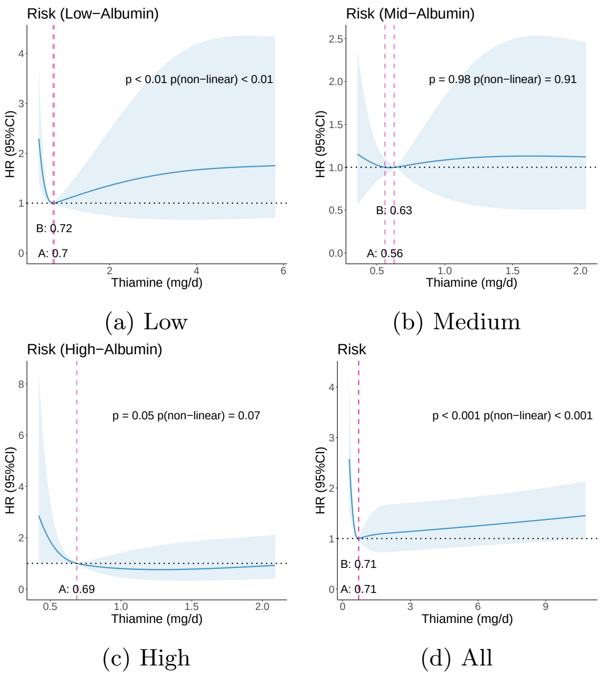

(a) Low (b) Medium

(c) High (d) All

Figure S21: Appropriate thiamine intake ranges for low mortality risk: [A, B] and [C, -)

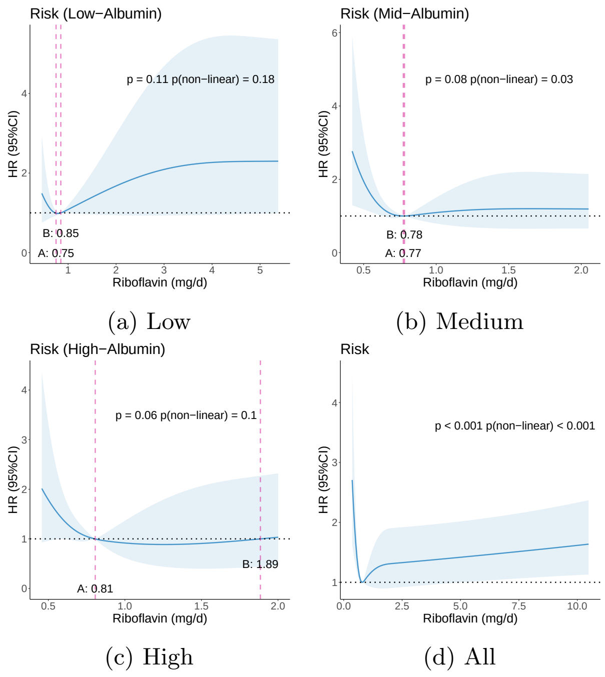

(a) Low (b) Medium

(c) High (d) All

Figure S22: Appropriate riboflavin intake ranges for low mortality risk: [A, B] and [C, -)

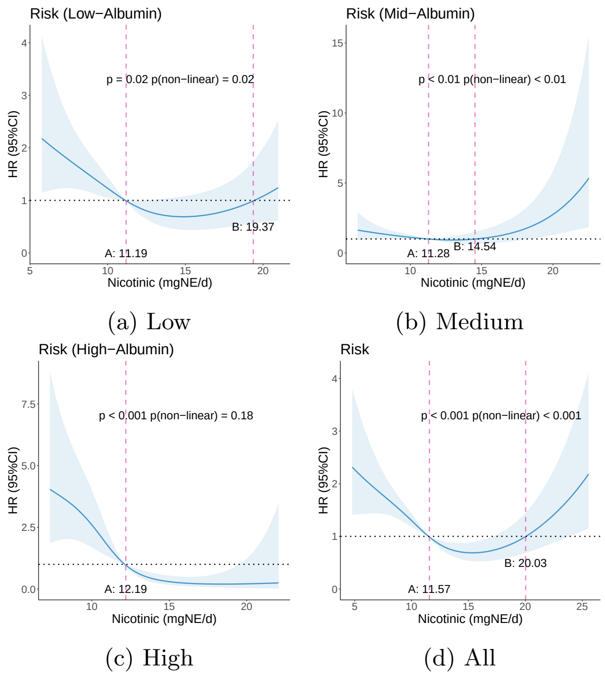

(a) Low (b) Medium

(c) High (d) All

Figure S23: Appropriate nicotinic intake ranges for low mortality risk: [A, B] and [C, -)

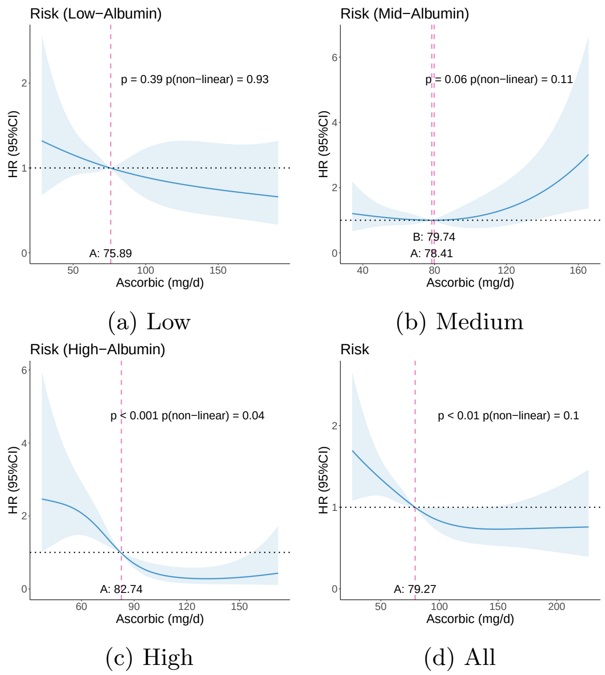

Figure S24: Appropriate ascorbic intake ranges for low mortality risk: [A, B] and [C, -)

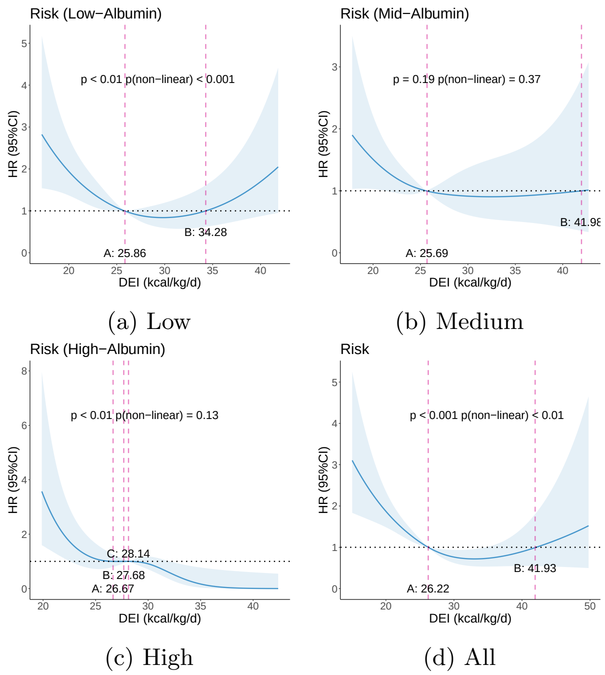

Figure S25: Appropriate DEI intake ranges for low mortality risk: [A, B] and [C, -)

E Appendix: Baseline Characteristics by Survival

Table S2: Baseline demographic characteristics and clinical laboratory findings by survival status[1].

		All		Survived		Deceased		
Variables	Unit	Mean	Std	Mean	Std	Mean	Std	P[2]
Age	year	59.11	15.78	62.72	15.09	55.48	15.64	¡0.001
Gender	-	female 327 (50%)		female 157 (48%)		female 170 (52%)		0.28
Height	cm	162.60	9.46	162.77	9.94	162.44	8.95	0.66
Weight	kg	61.17	11.95	60.29	12.23	62.05	11.61	0.06
BMI	-	23.19	5.89	22.92	7.43	23.46	3.74	0.24
Diab	-	diabetes 244 (37%)		diabetes 161 (49%)		diabetes 83 (25%)		¡0.001
SBP	mmHg	135.65	21.38	136.07	21.75	135.24	21.02	0.62
DBP	mmHg	78.97	14.20	76.57	14.92	81.39	13.02	¡0.001
WBC	$\times 10^9$/L	6.88	2.28	6.88	2.46	6.88	2.09	0.97
Hb	g/L	108.11	21.78	105.60	21.56	110.63	21.74	¡0.01
Urea	mmol/L	21.26	6.54	20.21	6.52	22.31	6.40	¡0.001
Scr	umol/L	720.38	254.87	648.18	239.98	793.02	249.03	¡0.001
K	mmol/L	4.33	0.78	4.26	0.84	4.40	0.70	0.02
Na	mmol/L	139.17	4.87	138.72	6.04	139.63	3.24	0.02
Cl	mmol/L	101.48	6.33	101.42	6.02	101.53	6.63	0.82
Ca	mmol/L	2.24	0.84	2.13	0.29	2.35	1.14	¡0.001
P	mmol/L	1.61	0.49	1.54	0.44	1.69	0.51	¡0.001
Hs-CRP	mg/L	5.66	13.29	7.19	16.62	4.12	8.50	¡0.01
Glucose	mmol/L	6.19	2.94	6.53	3.38	5.84	2.37	¡0.01
CO2CP	mmol/L	25.87	4.44	26.53	4.37	25.21	4.42	¡0.001
GFR	ml/min	0.06	0.60	0.09	0.85	0.03	0.02	0.23

Abbreviations: Cl, Chloride; CO2CP, Carbon Dioxide Combining Power; WBC, White Blood Cells; Hb, Hemoglobin; Urea; Ca, Calcium; K, Potassium; Na, Sodium; Scr, Serum Creatinine; P, Phosphorus; Hs-CRP, High Sensitivity C-Reactive Protein; Glucose; Weight; SBP, Systolic Blood Pressure; DBP, Diastolic Blood Pressure; Diab, Diabetes; BMI, Body Mass Index; GFR, Glomerular Filtration Rate.

[1] The study includes 656 patients, with 289 endpoint events of death recorded, and a total of 2,723 person-years of data.

[2] The term ”P” represents the overall test difference. The threshold for statistical significance was established at $P < 0.05$. In instances where $P < 0.01$ or $P < 0.001$, special symbols will denote these heightened levels of significance.

Table S3: Baseline dietary profiles by survival status[1].

		All		Survived		Deceased		
Variables	Unit	Mean	Std	Mean	Std	Mean	Std	P[2]
Water	g/d	1520.81	572.28	1493.35	568.83	1548.43	575.28	0.22
Protein	g/d	52.01	18.30	51.49	17.21	52.53	19.35	0.47
Fat	g/d	58.32	24.01	58.08	23.02	58.56	24.99	0.80
Carbohydrate	g/d	214.39	82.84	204.21	74.56	224.64	89.35	¡0.01
Calories	kcal/d	1544.92	523.92	1499.83	481.71	1590.28	560.28	0.03
Df	g/d	9.89	5.90	9.47	5.08	10.31	6.60	0.07
K	mg/d	1475.64	591.13	1454.35	594.16	1497.05	588.20	0.36
Na	mg/d	2307.36	1182.15	2403.03	1275.02	2211.10	1074.08	0.04
Mg	mg/d	246.99	98.91	243.91	96.17	250.09	101.65	0.42
Ca	mg/d	445.67	230.32	450.36	244.19	440.96	215.74	0.60
P	mg/d	792.38	283.65	785.43	263.10	799.37	303.16	0.53
Fe	mg/d	15.29	6.58	15.12	6.30	15.45	6.85	0.52
Zn	mg/d	7.87	3.33	7.88	3.15	7.86	3.50	0.95
Cu	mg/d	1.33	0.89	1.24	0.73	1.42	1.02	0.01
Mn	mg/d	3.93	1.86	3.77	1.71	4.09	1.99	0.03
Se	mg/d	36.77	21.34	35.66	15.84	37.88	25.69	0.18
Retinol	ug/d	166.30	207.21	171.70	170.04	160.87	238.99	0.50
VitaminA	ugRAE/d	463.23	392.35	460.42	394.39	466.05	390.86	0.85
Carotene	ug/d	1743.30	1978.37	1691.74	2071.80	1795.17	1881.45	0.50
VitaminE	mg/d	20.96	12.88	20.54	13.14	21.38	12.61	0.41
Thiamine	mg/d	1.66	10.08	1.78	10.90	1.54	9.21	0.76
Riboflavin	mg/d	1.73	9.85	1.90	10.77	1.56	8.85	0.67
Nicotinic	mg/d	11.98	6.00	11.90	5.44	12.06	6.51	0.72
Ascorbic	mg/d	88.58	72.44	83.78	70.05	93.42	74.56	0.09
DPI	g/(kg·d)	0.91	0.30	0.89	0.29	0.92	0.30	0.28
DEI	kcal/(kg·d)	26.93	8.58	26.04	8.35	27.83	8.73	¡0.01

Abbreviations: Df, Dietary Fiber; K, Potassium; Na, Sodium; Mg, Magnesium; Ca, Calcium; P, Phosphorus; Fe, Iron; Zn, Zinc; Cu, Copper; Mn, Manganese; Se, Selenium; Thiamine, Vitamin B1; Riboflavin, Vitamin B2; Nicotinic, Nicotinic Acid; Ascorbic, Ascorbic Acid; DPI, Dietary Protein Intake; DEI, Dietary Energy Intake.

[1] The study includes 656 patients, with 289 endpoint events of death recorded, and a total of 2,723 person-years of data.

[2] The term "P" represents the overall test difference. The threshold for statistical significance was established at $P < 0.05$. In instances where $P < 0.01$ or $P < 0.001$, special symbols will denote these heightened levels of significance.
